# Sociodemographic and clinicopathologic features associated with rapid relapse in triple-negative breast cancer: a Swedish nationwide registry-based study

**DOI:** 10.1007/s10549-026-08115-z

**Published:** 2026-10-28

**Authors:** Sithumini Sarathchandra, Jenny Nyqvist-Streng, Elin Sahlberg, Zakaria Einbeigi, Khalil Helou, Anikó Kovács, Slavica Janeva, Per Karlsson, Toshima Z. Parris

**Affiliations:** 1 Department of Oncology, Institute of Clinical Sciences, Sahlgrenska Academy, University of Gothenburg, Gothenburg, Sweden; 2 Sahlgrenska Center for Cancer Research, Sahlgrenska Academy, University of Gothenburg, Gothenburg, Sweden; 3 Department of Surgery, Skaraborg Hospital, Region Västra Götaland, Skövde, Sweden; 4 Department of Surgery, Institute of Clinical Sciences, Sahlgrenska Academy, University of Gothenburg, Gothenburg, Sweden; 5 Department of Oncology, Sahlgrenska University Hospital, Region Västra Götaland, Gothenburg, Sweden; 6 Department of Oncology, Borås Hospital, Region Västra Götaland, Borås, Sweden; 7 Department of Clinical Pathology, Sahlgrenska University Hospital, Region Västra Götaland, Gothenburg, Sweden; 8 Department of Surgery, Sahlgrenska University Hospital, Region Västra Götaland, Gothenburg, Sweden

**Keywords:** Health disparities, Tumor aggressiveness, Risk stratification, Prognostic biomarkers, Recurrence patterns, Treatment response

## Abstract

**Purpose:**

To characterize clinicopathologic, treatment-related, and sociodemographic factors associated with rapid versus late relapse in triple-negative breast cancer (TNBC), and to evaluate how relapse timing influences metastatic patterns and survival outcomes.

**Methods:**

Using linked Swedish population-based registers, we conducted a retrospective cohort study of women diagnosed with non-metastatic TNBC between 2007 and 2021. Patients were classified into three groups based on time to distant relapse or death: rapid relapse (rrTNBC; ≤24 months), late relapse (lrTNBC; >24 months), or event-free at last follow-up. Tumor characteristics, comorbidity, treatments, and sociodemographic variables were compared across groups. Multivariable logistic regression and survival outcomes were assessed. Sensitivity analyses restricted to healthcare regions with complete relapse data were performed.

**Results:**

Among 8,298 patients, 15% experienced rrTNBC, 18% lrTNBC, and 67% remained event-free. rrTNBC was associated with older age, larger tumors, nodal involvement, advanced stage, and a higher frequency of visceral and multi-organ metastases. Patients with rrTNBC were less likely to receive adjuvant systemic therapy or radiotherapy and more likely to undergo mastectomy. Sociodemographic disparities were observed, with rrTNBC more frequent among patients who were single or widowed, lower educated, or had lower household income. Survival outcomes were substantially worse for rrTNBC compared with lrTNBC and event-free patients.

**Conclusions:**

Rapid relapse defines a particularly vulnerable TNBC population characterized by aggressive tumor biology, early dissemination, inferior survival, and sociodemographic disadvantage, while lrTNBC represents an intermediate phenotype with delayed disease progression. Distinguishing rrTNBC from lrTNBC may improve risk stratification, guide treatment decisions, and support more personalized follow-up strategies.

**Supplementary Information:**

The online version contains supplementary material available at https://doi.org/10.1007/s10549-026-08115-z.

## Introduction

Triple-negative breast cancer (TNBC) is one of the most lethal and therapeutically challenging breast cancer subtypes, defined by the lack of hormone receptors and HER2 amplification/overexpression, while also exhibiting clinical and biological heterogeneity, high proliferative activity, and limited therapeutic options [1–3]. Although most patients with early-stage TNBC are cured, about 30% of patients in historical cohorts develop recurrent or metastatic disease within three years of diagnosis [4, 5]. Prognosis remains poor for those who develop metastatic relapse, with a reported median survival of approximately 14 months [6]. The risk of recurrence and death is highest during the first five years after diagnosis [7–9]. Other cohort studies report a median time to distant metastasis of approximately two years [5, 10]. Consequently, rapid relapse TNBC (rrTNBC) is commonly defined as distant recurrence or death within 24 months of diagnosis [5, 11–13]. In contrast to rrTNBC, a smaller but clinically relevant subgroup of patients experience late relapse, with distant recurrence or death occurring beyond the initial high-risk period [14–16]. Recent studies suggest that late relapse TNBC (lrTNBC) may represent a biologically distinct phenotype with reduced aggressiveness [14]. The temporal stratification of relapse therefore offers important insight into TNBC heterogeneity.

Evaluation of tumor recurrence should consider both tumor- and patient-related factors. Established clinicopathologic features (e.g., tumor stage, histological grade, lymph node status, and Ki67 proliferative activity) are well-known prognostic factors in TNBC, whereas sociodemographic characteristics (e.g., race, income, and education) are associated with stage at diagnosis and outcomes though their independent effect on relapse timing after adjustment for tumor biology remains unclear [7, 11, 17, 18]. Beyond tumor-intrinsic characteristics, patient-related factors such as comorbidity burden and sociodemographic features have been linked to rrTNBC [11, 14, 19]. Higher comorbidity burden, commonly assessed using the Charlson comorbidity index, is associated with an increased risk of rapid relapse, potentially reflecting both competing health risks and reduced tolerance to intensive multimodal therapy [19–21]. Patients with higher comorbidity indices are also less likely to receive guideline-concordant treatment [20, 22–24], including surgery and systemic chemotherapy, which is associated with more unfavorable relapse-free outcomes in TNBC [19]. Similarly, socioeconomic disadvantage (e.g., lower income, lack of private insurance or reliance on public insurance, and unmarried status) has been associated with higher odds of rrTNBC, even after adjustment for tumor stage, particularly in settings with unequal access to care [11, 13, 19].

The temporal stratification of TNBC relapse offers an opportunity to distinguish aggressive tumor biology from patient- and sociodemographic factors. In retrospective, population-based cohort study, we use Swedish registry data to systematically characterize clinical, pathological, and sociodemographic factors associated with rrTNBC and lrTNBC that can refine risk stratification and inform more personalized management strategies for this heterogeneous disease.

## Methods

### Study population and data

To determine the sociodemographic and clinicopathologic features associated with rapid relapse in TNBC (estrogen receptor-negative [ER-], progesterone receptor-negative [PR-], HER2-negative [HER2-], either at baseline or after surgery), population-based registry data were retrospectively obtained for 9262 women diagnosed with invasive TNBC (either at baseline or after surgery) in Sweden between 2007 and 2021, as described elsewhere [20]. In brief, data were sourced from multiple Swedish registers: the National Breast Cancer Register (NBCR; data accessed 2023 and 2024), Swedish Patient Register (SPR; data accessed 2023), Swedish Cause of Death Register (SCDR; data accessed 2023), and Statistics Sweden (SCB; data accessed 2024). Data from multiple registers were linked via the personal identity number assigned to all Swedish residents. NBCR provided detailed information on the primary tumor, patient demographics, treatment, and relapse. Relapse information was available from four of six Swedish healthcare regions (Stockholm/Gotland, South, Southeast, and West), while survival data were available nationwide. Established in 2008, NBCR serves as a tool for quality assurance, benchmarking, and research, maintaining a high level of data completeness, comparability, validity, and timely data reporting [25].

**Fig. 1:**
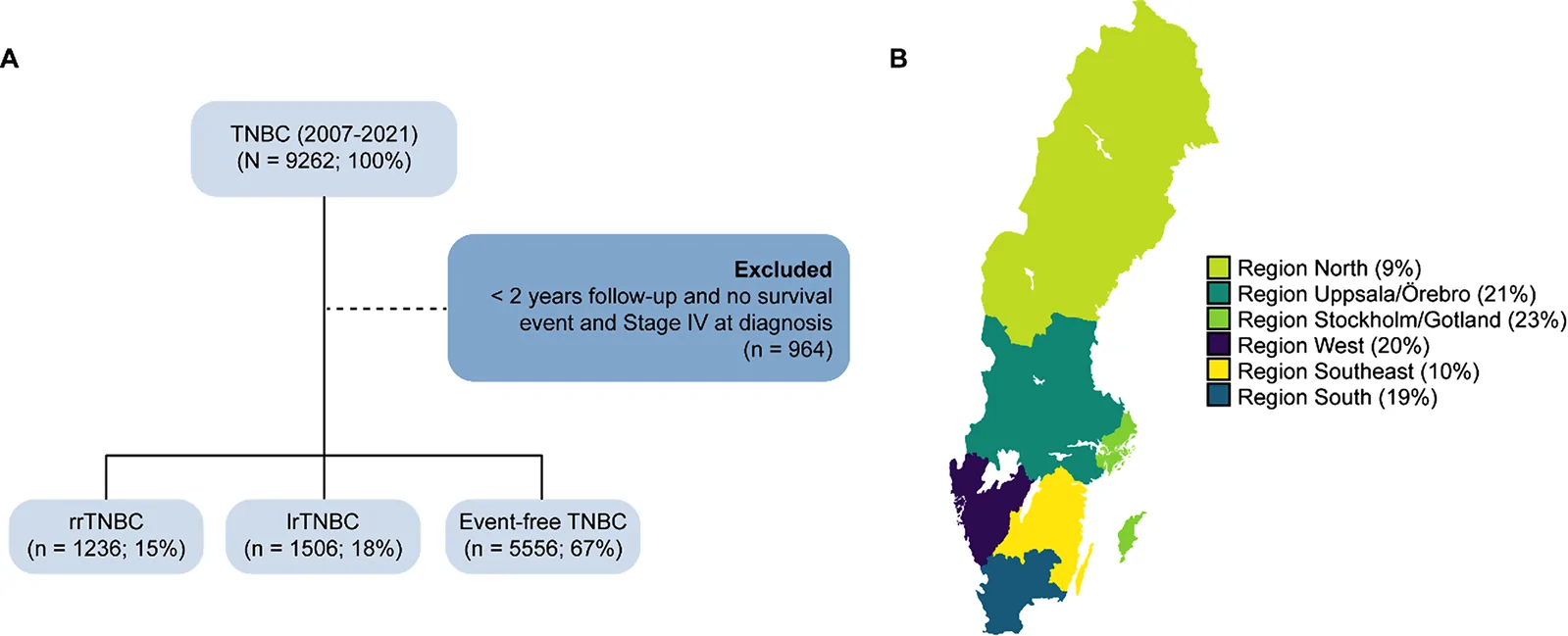
Study overview. (**A**) According to Swedish registry data, 9262 patients were diagnosed with triple-negative breast cancer (TNBC) between 2007 and 2021. Patients with < 2 years of follow-up and no survival event, and those with stage IV at diagnosis were excluded (*n* = 964). The remaining 8298 patients were classified into 3 groups: rapid relapse or death ≤ 24 months after diagnosis (rrTNBC; *n* = 1236), late relapse or death > 24 months after diagnosis (lrTNBC; *n* = 1506), and event-free at last follow-up (event-free TNBC; *n* = 5556). (**B**) Map showing the six healthcare regions in Sweden, with the percentage of TNBC patients included in the study within parentheses

Information on comorbidities was obtained from the SPR, and the cause of death was obtained from the SCDR. The weighted Charlson comorbidity index (CCIw), a measure of the burden of comorbidities [26], was calculated using in- and outpatient information retrieved from the SPR regarding various diseases (i.e., myocardial infarction, congestive heart failure, peripheral vascular disease, cerebrovascular disease, pulmonary diseases, rheumatic disease, dementia, hemiplegia, diabetes, chronic kidney disease, liver disease, gastric peptic ulcer disease, cancer, and HIV/AIDS). SCB provided sociodemographic data, including country of birth, household income, marital status, and educational level. The exclusion criteria were (1) < 2 years follow-up and no survival event and (2) stage IV disease at diagnosis (Fig. 1A). For cases with bilateral breast cancer, only the first detected tumor was included. The end of follow-up was set to the date or death or the administrative cutoff date (January 1, 2023), whichever occurred first. All procedures were done in accordance with the Declaration of Helsinki and approved by the Central Ethical Review Board and the Swedish Ethical Review Authority (reference numbers 2019–05676; 2021–04421; 2022-02946-02; 2023-07905-02).

### Outcomes and definitions

According to Swedish national guidelines, tumors with ER and PR expression < 10% were classified as negative. Ki67 expression was categorized as low (< 20%) or high (≥ 20%). HER2 status was defined as negative in cases with HercepTest scores of 0 or 1+, or 2 + with no HER2 gene amplification by SISH [27]. The 8298 patients included in the study were categorized into three relapse groups based on the time to relapse or death: (1) rapid relapse or death ≤ 24 months (rrTNBC; *n* = 1236), (2) late relapse or death > 24 months (lrTNBC; *n* = 1506), and (3) event-free at last follow-up (event-free TNBC; *n* = 5556; Fig. 1A). Rapid relapse or death within 24 months was selected a priori based on previous rrTNBC studies applying the same two-year threshold [5, 11, 13, 19], thereby facilitating comparison with the existing literature. Patients classified as event-free had no recorded distant relapse or death by the end of their available follow-up and were required to have at least 24 months of follow-up.

### Statistical analysis

R/Bioconductor (R version 4.4.1) was utilized for statistical analysis, with a significance level of 0.05 and two-sided p-values. The tableone package (version 0.13.2) in R was used to calculate p-values using the Chi-square test (with continuity correction) for categorical variables and ANOVA for continuous variables [28]. Continuous variables were summarized as mean (standard deviation [SD]) or median (interquartile range [IQR]), as appropriate based on their distributions. Median follow-up was estimated using the reverse Kaplan–Meier method, with follow-up calculated from the date of diagnosis to death or last known follow-up using the survival R package (version 3.8-3). Using the ggsurvfit R package (version 1.1.0), survival rates were assessed using the log-rank test and depicted using Kaplan-Meier curves and multivariable Cox regression models adjusted for time to relapse or death, patient age, postoperative tumor size, postoperative tumor grade, axillary lymph node status, CCIw, and adjuvant chemotherapy received. Cumulative incidence curves for competing risks were generated with the ggcompetingrisks function in the R survminer package (version 0.4.9) [29]. Smoothed hazard functions were estimated with the muhaz R package (version 1.2.6.4).

Logistic regression was conducted to assess the association between relapse or death group with clinical, pathological, and sociodemographic variables using the caret package (version 6.0–94). To assess the association between relapse or death group and treatment modality (BCS and ART vs. other, mastectomy vs. other, ACT vs. no ACT, NACT vs. no NACT) within each age group, multivariable logistic regression models were fitted and adjusted for tumor size, Nottingham histological grade (NHG), axillary lymph node status, and relapse or death group. Odds ratios (ORs) with 95% confidence intervals (CIs) were estimated. The time interval between initial diagnosis and death from breast cancer (disease-specific survival; DSS), the time interval between initial diagnosis and death from any cause (overall survival; OS), and the time interval between initial diagnosis and the first occurrence of disease recurrence (local, regional, or distant) or death from any cause, whichever occurred first (disease-free survival; DFS) were used to define patient survival rates.

The primary analyses included the full study population, with cumulative incidence and survival outcomes calculated from the date of breast cancer diagnosis. A first sensitivity analysis was restricted to patients treated in the four healthcare regions (Stockholm/Gotland, South, Southeast, and West) with available distant metastasis data, using the same definitions and clinical endpoints (OS, DSS, and DFS) as the primary analyses. In a second sensitivity analysis, analyses were restricted to patients with a documented date of distant metastasis. In this subgroup, post-metastasis overall survival was calculated from the date of distant metastasis to death from any cause or last follow-up, and post-metastasis disease-specific survival was calculated from the date of distant metastasis to breast cancer-related death. Thirty-eight patients with a reported distant metastasis after the administrative cutoff date were excluded from this analysis.

## Results

### Patient and tumor characteristics

Of 9262 patients diagnosed with TNBC in Sweden between 2007 and 2021, 964 patients with less than 2 years follow-up and no survival event (*n* = 701) and stage IV disease at diagnosis (*n* = 263) were excluded (Fig. 1A). The remaining 8298 patients were classified into three groups to evaluate factors associated with the time to distant relapse or death: rapid relapse or death within 24 months of diagnosis (rrTNBC; *n* = 1236; 15%), late relapse or death > 24 months (lrTNBC; *n* = 1506; 18%), and event-free at last follow-up (event-free; *n* = 5556; 67%). Median follow-up time was 8.2 years (95% CI, 8.0-8.3) for the full cohort, 6.6 years for patients with rrTNBC, 15.8 years for those with lrTNBC, and 7.6 years for event-free patients. Most patients were diagnosed in densely populated healthcare regions, including Stockholm/Gotland (23%), Uppsala/Örebro (21%), West (20%), and South (19%; Fig. 1B). The median age at diagnosis was 62 years (IQR, 49–72 years) and most patients presented with clinical stage I-II disease (*n* = 7185; 87%) and pathological stage I-II disease (*n* = 5965; 72%), had grade 3 tumors (*n* = 5504; 66%), and were classified as TNBC of no special type (TNBC-NST; *n* = 5883; 71%). The cohort exhibited a high mean postoperative Ki67 (mean, 57%; SD, 27%), and most patients had no reported comorbidities (*n* = 5704; 69%; Table 1). Patients in the rrTNBC group were significantly older than those in the lrTNBC and event-free groups (median age, rrTNBC 71 years, lrTNBC 69 years, event-free 59 years), with a higher proportion aged ≥ 75 years of age. Additionally, they also presented with more aggressive tumor features, i.e., larger tumors at diagnosis (median: rrTNBC 40 mm, lrTNBC 37 mm, event-free 28 mm) and after surgery (median: rrTNBC 30 mm, lrTNBC 22 mm, event-free 18 mm), and stage III disease at diagnosis (rrTNBC 19%, lrTNBC 7%, non-rrTNBC 3%) and after surgery (rrTNBC 28%, lrTNBC 14%, event-free 5%).

**Table 1:** Patient and tumor characteristics of triple-negative breast cancer patients (*n* = 8298), stratified by time to relapse or death

Characteristic	Overall (*n* = 8298)	Rapid relapse or death (*n* = 1236)	Late relapse or death (*n* = 1506)	Event-free at last follow-up (*n* = 5556)	*P*
**Swedish region (%)**					**0.026**
North	729 (8.8)	95 (7.7)	158 (10.5)	476 (8.6)	
Stockholm/Gotland	1870 (22.5)	303 (24.5)	309 (20.5)	1258 (22.6)	
South	1534 (18.5)	224 (18.1)	267 (17.7)	1043 (18.8)	
Southeast	825 (9.9)	127 (10.3)	153 (10.2)	545 (9.8)	
Uppsala/Örebro	1723 (20.8)	236 (19.1)	299 (19.9)	1188 (21.4)	
West	1617 (19.5)	251 (20.3)	320 (21.2)	1046 (18.8)	
**Age**,** years**,** median [IQR]**	62.0 [49.0, 72.0]	71.0 [54.0, 82.0]	69.0 [58.0, 79.0]	59.0 [47.0, 68.0]	**< 0.001**
**Age group**,** years (%)**					**< 0.001**
< 40	801 (9.7)	93 (7.5)	86 (5.7)	622 (11.2)	
40–49	1279 (15.4)	143 (11.6)	141 (9.4)	995 (17.9)	
50–64	2654 (32.0)	233 (18.9)	364 (24.2)	2057 (37.0)	
65–74	1904 (22.9)	220 (17.8)	350 (23.2)	1334 (24.0)	
≥ 75	1660 (20.0)	547 (44.3)	565 (37.5)	548 (9.9)	
**Clinical stage (%)**					**< 0.001**
0	59 (0.7)	1 (0.1)	4 (0.3)	54 (1.0)	
IA	3005 (36.2)	178 (14.4)	442 (29.3)	2385 (42.9)	
IIA	2982 (35.9)	428 (34.6)	604 (40.1)	1950 (35.1)	
IIB	1198 (14.4)	345 (27.9)	241 (16.0)	612 (11.0)	
IIIA	303 (3.7)	129 (10.4)	58 (3.9)	116 (2.1)	
IIIB	146 (1.8)	86 (7.0)	33 (2.2)	27 (0.5)	
IIIC	52 (0.6)	21 (1.7)	11 (0.7)	20 (0.4)	
Unspecified	553 (6.7)	48 (3.9)	113 (7.5)	392 (7.1)	
**Tumor size**,** mm**, median [IQR]*	30.0 [22.0, 41.8]	40.0 [26.3, 53.8]	36.5 [31.8, 44.0]	28.0 [22.0, 37.3]	**< 0.001**
Ki67% (mean (SD))*	63.8 (23.9)	62.3 (24.6)	58.8 (25.8)	65.2 (23.1)	**0.002**
**Pathological stage (%)†**					**< 0.001**
0	26 (0.3)	1 (0.1)	0 (0.0)	25 (0.4)	
IA	2631 (31.7)	129 (10.4)	336 (22.3)	2166 (39.0)	
IIA	2382 (28.7)	236 (19.1)	456 (30.3)	1690 (30.4)	
IIB	952 (11.5)	190 (15.4)	211 (14.0)	551 (9.9)	
IIIA	567 (6.8)	214 (17.3)	151 (10.0)	202 (3.6)	
IIIC	257 (3.1)	138 (11.2)	62 (4.1)	57 (1.0)	
Missing data	1483 (17.9)	328 (26.5)	290 (19.3)	865 (15.6)	
**Cancer type (%)†**					**< 0.001**
TNBC-NST	5883 (70.9)	771 (62.4)	1120 (74.4)	3992 (71.9)	
Other invasive cancer	975 (11.7)	118 (9.5)	162 (10.8)	695 (12.5)	
TNBC-ILC	165 (2.0)	35 (2.8)	45 (3.0)	85 (1.5)	
Both TNBC-NST and TNBC-ILC	32 (0.4)	6 (0.5)	4 (0.3)	22 (0.4)	
TNBC-NST and other type than TNBC-ILC	99 (1.2)	12 (1.0)	14 (0.9)	73 (1.3)	
Missing data	1144 (13.8)	294 (23.8)	161 (10.7)	689 (12.4)	
**Tumor size**,** mm**,** median [IQR]†**	20.0 [13.0, 29.0]	30.0 [20.0, 44.0]	22.0 [15.0, 32.0]	18.0 [11.0, 25.0]	**< 0.001**
**NHG (%)†**					**< 0.001**
Grade 1	167 (2.0)	9 (0.7)	32 (2.1)	126 (2.3)	
Grade 2	1381 (16.6)	137 (11.1)	293 (19.5)	951 (17.1)	
Grade 3	5504 (66.3)	782 (63.3)	997 (66.2)	3725 (67.0)	
**Ki67% (mean (SD))†**	57.1 (27.0)	61.6 (25.3)	55.1 (26.5)	56.6 (27.4)	**< 0.001**
**Overall follow-up time**,** years (mean (SD))**	6.6 (4.2)	1.5 (1.0)	5.5 (3.0)	8.0 (3.9)	**< 0.001**
**Time to distant relapse or death**,** years (mean (SD))**	6.5 (4.2)	1.1 (0.5)	5.3 (3.0)	8.0 (3.9)	**< 0.001**
**Number of distant metastatic sites (%)**					**< 0.001**
0	7560 (91.1)	776 (62.8)	1228 (81.5)	5556 (100.0)	
1	456 (5.5)	292 (23.6)	164 (10.9)	0 (0.0)	
≥ 2	282 (3.4)	168 (13.6)	114 (7.6)	0 (0.0)	
**Comorbidities (%)**					
Myocardial infarction	191 (2.3)	73 (5.9)	54 (3.6)	64 (1.2)	**< 0.001**
Congestive heart failure	172 (2.1)	73 (5.9)	48 (3.2)	51 (0.9)	**< 0.001**
Peripheral vascular disease	147 (1.8)	49 (4.0)	39 (2.6)	59 (1.1)	**< 0.001**
Cerebrovascular disease	453 (5.5)	128 (10.4)	118 (7.8)	207 (3.7)	**< 0.001**
Chronic obstructive pulmonary disease	141 (1.7)	41 (3.3)	46 (3.1)	54 (1.0)	**< 0.001**
Chronic other pulmonary disease	268 (3.2)	45 (3.6)	62 (4.1)	161 (2.9)	**0.04**
Rheumatic disease	359 (4.3)	70 (5.7)	79 (5.2)	210 (3.8)	**0.002**
Dementia	67 (0.8)	39 (3.2)	17 (1.1)	11 (0.2)	**< 0.001**
Hemiplegia	26 (0.3)	5 (0.4)	5 (0.3)	16 (0.3)	0.794
Diabetes without chronic complication	8 (0.1)	2 (0.2)	1 (0.1)	5 (0.1)	0.7
Diabetes with chronic complication	131 (1.6)	32 (2.6)	39 (2.6)	60 (1.1)	**< 0.001**
Renal disease	69 (0.8)	28 (2.3)	12 (0.8)	29 (0.5)	**< 0.001**
Mild liver disease	66 (0.8)	15 (1.2)	21 (1.4)	30 (0.5)	**0.001**
Liver special	5 (0.1)	1 (0.1)	1 (0.1)	3 (0.1)	0.936
Severe liver disease	9 (0.1)	0 (0.0)	5 (0.3)	4 (0.1)	**0.011**
Peptic ulcer disease	141 (1.7)	31 (2.5)	46 (3.1)	64 (1.2)	**< 0.001**
Malignancy	1169 (14.1)	214 (17.3)	295 (19.6)	660 (11.9)	**< 0.001**
Metastatic solid tumor	73 (0.9)	21 (1.7)	20 (1.3)	32 (0.6)	**< 0.001**
AIDS	3 (0.0)	1 (0.1)	1 (0.1)	1 (0.0)	0.455
**CCIw group (%)**					**< 0.001**
CCIw = 0	5704 (68.7)	684 (55.3)	859 (57.0)	4161 (74.9)	
CCIw = 1–3	2349 (28.3)	462 (37.4)	578 (38.4)	1309 (23.6)	
CCIw = 4–10	245 (3.0)	90 (7.3)	69 (4.6)	86 (1.5)	

Only patients with invasive disease (either at diagnosis or after surgery) were included. Abbreviations: CCIw, weighted Charlson comorbidity index; TNBC, Triple-negative breast cancer; TNBC–ILC, Triple-negative breast cancer–invasive lobular cancer; TNBC–NST, Triple-negative breast cancer of no special type. Statistically significant differences (*P* < 0.05) are highlighted in bold. P-values were calculated using Chi-square test for categorical variables (with continuity correction) and ANOVA for continuous variables

**Fig. 2:**
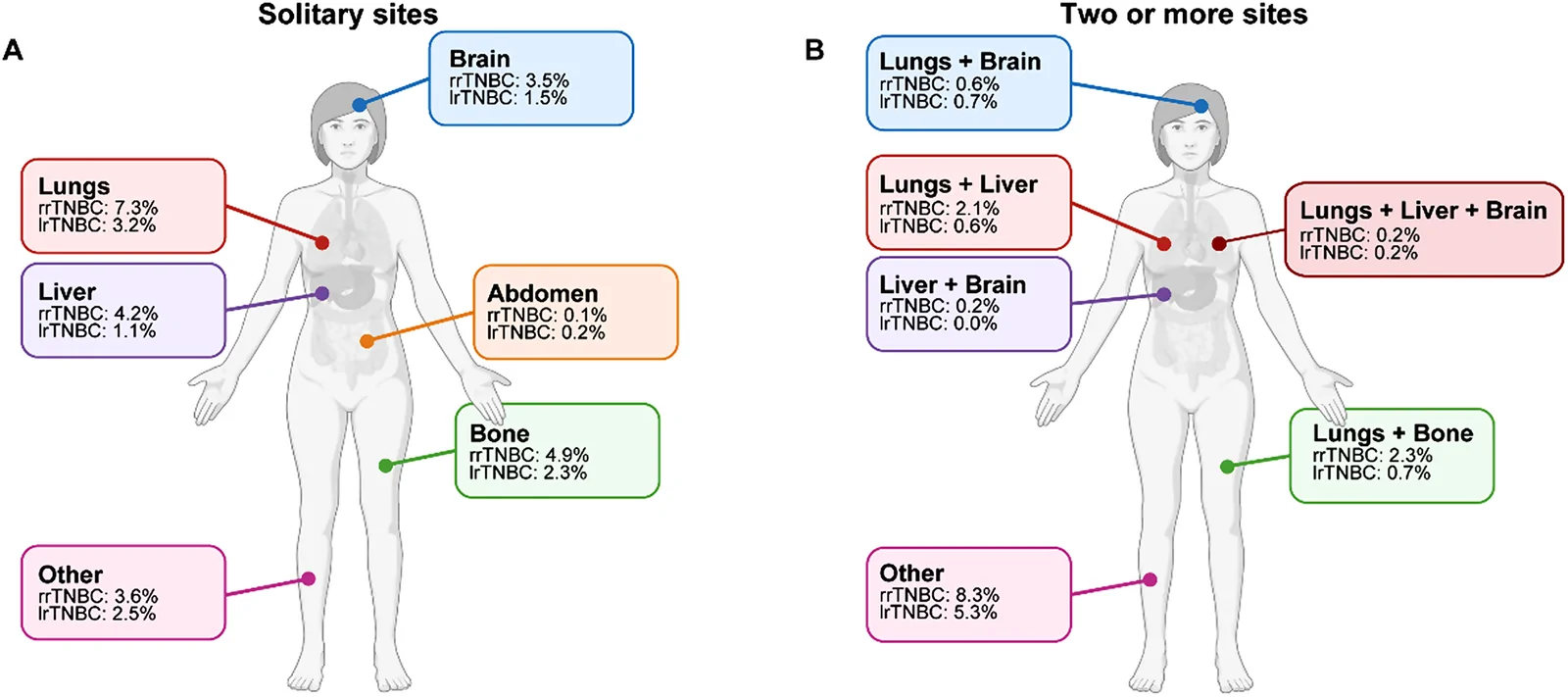
Distribution of distant metastatic sites in rapid relapse or death (rrTNBC) and late relapse or death (lrTNBC). (**A**) Proportion of patients with solitary metastatic sites (abdomen, bone, brain, liver, lungs, and other) in rrTNBC and lrTNBC. (**B**) Proportion of patients with multiple-site (≥ 2 sites) metastatic involvement, showing common organ combinations, in rrTNBC and lrTNBC. Percentages indicate the frequency within each group. Created in BioRender

Distant relapse was reported in 742 patients (rrTNBC: *n* = 463; lrTNBC: *n* = 279), with the rrTNBC group exhibiting a greater metastatic burden and broader organ involvement compared with lrTNBC. In total, 456 patients (5.5%) had a solitary metastatic site, 192 (2.3%) had 2 sites, and 90 (1.1%) had 3 or more sites; metastatic site information was missing for four patients (Table 1). The mean time to distant relapse or death was 13 months (SD, 6.5) in rrTNBC, 64 months (SD, 36) in lrTNBC, and 96 months (SD, 47) in the event-free group. The most common solitary metastatic sites were bone, brain, liver, and lungs, with rrTNBC showing approximately 2-3-fold higher proportions than lrTNBC across all organs (Fig. 2). Abdominal metastases were rare in both groups. The most common two-site metastases involved the lungs+bone or lungs+liver, whereas combinations involving the brain (e.g., lungs+brain, liver+brain) were uncommon. rrTNBC consistently showed higher proportions than lrTNBC across all metastatic patterns. However, multi-organ involvement of three or more organs (e.g., lungs+liver+brain) was rare. Regarding CNS metastases, 128 patients were identified, of whom 75 (58.6%) had rrTNBC and 53 (41.4%) had lrTNBC. Patients with rrTNBC had a more advanced clinical-stage distribution (*P* = 0.005), higher mean preoperative Ki-67 (66.2% vs. 54.3%; *P* = 0.040), and larger median pathological tumor size (23.5 vs. 18.0 mm; *P* = 0.007). No statistically significant differences in treatment or sociodemographic characteristics were observed between the groups (Supplementary Tables S2–S4).

### rrTNBC linked to more radical surgery, less adjuvant therapy, and lower socioeconomic status

We then evaluated the association between administered treatment, sociodemographic features, and relapse or death in the patient cohort. Patients who remained event-free at follow-up were more likely to receive breast-conserving surgery (BCS), sentinel lymph-node biopsy, and anthracycline+taxane neoadjuvant and adjuvant chemotherapy (NACT and ACT), along with adjuvant radiotherapy (ART) and bisphosphonate therapy (Table 2). In contrast, the rrTNBC and lrTNBC groups had higher rates of mastectomy and axillary dissection, and were less likely to receive ACT or ART. However, the high degree of missingness for chemotherapy may limit generalizability. Multivariable logistic regression analysis demonstrated that, with the exception of rrTNBC patients < 40 years of age, rrTNBC and lrTNBC patients across all age groups were less likely to receive BCS + ART (Table 3). In contrast, patients aged ≥ 50 years in both groups were more likely to undergo mastectomy but were less likely to be given ACT. The age threshold for NACT administration was 65 years: rrTNBC patients aged < 65 years (in both groups) were more likely to receive NACT, while lrTNBC patients aged ≥ 65 years were less likely to receive NACT.

**Table 2:** Treatment characteristics of triple-negative breast cancer patients (*n* = 8298), stratified by time to relapse or death

Characteristic	Overall (*n* = 8298)	Rapid relapse or death (*n* = 1236)	Late relapse or death (*n* = 1506)	Event-free at last follow-up (*n* = 5556)	*P*
**NACT type (%)**					**< 0.001**
No NACT	26 (0.3)	6 (0.5)	14 (0.9)	6 (0.1)	
Anthracycline-based	270 (3.3)	71 (5.7)	52 (3.5)	147 (2.6)	
Anthracycline+taxane	1119 (13.5)	176 (14.2)	106 (7.0)	837 (15.1)	
Taxane-based	75 (0.9)	25 (2.0)	7 (0.5)	43 (0.8)	
Other	30 (0.4)	10 (0.8)	5 (0.3)	15 (0.3)	
Missing data	6778 (81.7)	948 (76.7)	1322 (87.8)	4508 (81.1)	
**Axillary surgery type (%)**					**< 0.001**
SLNB	4843 (58.4)	315 (25.5)	717 (47.6)	3811 (68.6)	
AD	1986 (23.9)	550 (44.5)	436 (29.0)	1000 (18.0)	
Sampling (including SLNB)	113 (1.4)	15 (1.2)	29 (1.9)	69 (1.2)	
SLNB + AD	938 (11.3)	139 (11.2)	213 (14.1)	586 (10.5)	
**Surgery type (%)**					**< 0.001**
Only axillary surgery	29 (0.3)	3 (0.2)	2 (0.1)	24 (0.4)	
Breast-conserving surgery	4274 (51.5)	288 (23.3)	579 (38.4)	3407 (61.3)	
Mastectomy	3752 (45.2)	804 (65.0)	890 (59.1)	2058 (37.0)	
Subcutaneous mastectomy	61 (0.7)	6 (0.5)	3 (0.2)	52 (0.9)	
Missing data	182 (2.2)	135 (10.9)	32 (2.1)	15 (0.3)	
**Locoregional_treatment (%)**					**< 0.001**
BCS + ART	3407 (41.1)	161 (13.0)	385 (25.6)	2861 (51.5)	
Mx-ART	1521 (18.3)	274 (22.2)	402 (26.7)	845 (15.2)	
Mx + ART	1450 (17.5)	311 (25.2)	293 (19.5)	846 (15.2)	
Other	1920 (23.1)	490 (39.6)	426 (28.3)	1004 (18.1)	
**ACT type (%)**					**< 0.001**
No ACT	2419 (29.2)	444 (35.9)	574 (38.1)	1401 (25.2)	
Anthracycline-based	1784 (21.5)	120 (9.7)	316 (21.0)	1348 (24.3)	
Anthracycline+taxane	1777 (21.4)	101 (8.2)	144 (9.6)	1532 (27.6)	
Taxane-based	216 (2.6)	49 (4.0)	20 (1.3)	147 (2.6)	
Other	556 (6.7)	113 (9.1)	114 (7.6)	329 (5.9)	
Missing data	1546 (18.6)	409 (33.1)	338 (22.4)	799 (14.4)	
**ART (%)**	4929 (59.4)	485 (39.2)	692 (45.9)	3752 (67.5)	**< 0.001**
**Adjuvant bisphosphonate therapy (%)**	915 (11.0)	79 (6.4)	105 (7.0)	731 (13.2)	**< 0.001**

Abbreviations: ACT, adjuvant chemotherapy; AD, axillary dissection; ART, adjuvant radiotherapy; BCS, breast-conserving surgery; Mx, mastectomy; NACT, neoadjuvant chemotherapy; SLNB, sentinel lymph-node biopsy. Statistically significant differences (*P* < 0.05) are highlighted in bold. P-values were calculated using Chi-square test for categorical variables (with continuity correction) and ANOVA for continuous variables

**Table 3:** Multivariable logistic regression analysis with odds ratio and 95% confidence intervals based on treatment modality and relapse or death group

	Age group				
	< 40 years	40–49 years	50–64 years	65–75 years	≥ 75 years
	OR (95% CI)	OR (95% CI)	OR (95% CI)	OR (95% CI)	OR (95% CI)
**BCS and ART vs. other**					
Relapse or death group					
Event-free	1.00	1.00	1.00	1.00	1.00
lrTNBC	**0.53 (0.27**,** 0.98)**	**0.54 (0.35**,** 0.82)**	**0.46 (0.36**,** 0.60)**	**0.41 (0.32**,** 0.54)**	**0.39 (0.28**,** 0.54)**
rrTNBC	0.49 (0.21, 1.04)	**0.36 (0.21**,** 0.61)**	**0.40 (0.27**,** 0.58)**	**0.25 (0.16**,** 0.38)**	**0.20 (0.12**,** 0.32)**
**Mastectomy vs. other surgery**					
Relapse or death group					
Event-free	1.00	1.00	1.00	1.00	1.00
lrTNBC	1.12 (0.64, 1.97)	1.33 (0.86, 2.04)	**1.83 (1.39**,** 2.39)**	**1.60 (1.23**,** 2.09)**	**1.72 (1.29**,** 2.32)**
rrTNBC	1.51 (0.77, 3.03)	1.43 (0.87, 2.34)	**1.78 (1.23**,** 2.58)**	**1.92 (1.32**,** 2.79)**	**1.76 (1.25**,** 2.50)**
**ACT vs. no ACT**					
Relapse or death group					
Event-free	1.00	1.00	1.00	1.00	1.00
lrTNBC	0.56 (0.32, 1.03)	0.58 (0.38, 0.89)	**0.53 (0.41**,** 0.69)**	**0.41 (0.31**,** 0.53)**	**0.35 (0.25**,** 0.48)**
rrTNBC	0.62 (0.31, 1.26)	0.64 (0.39, 1.06)	**0.35 (0.25**,** 0.50)**	**0.32 (0.22**,** 0.46)**	**0.23 (0.16**,** 0.34)**
**NACT vs. no NACT**					
Relapse or death group					
Event-free	1.00	1.00	1.00	1.00	1.00
lrTNBC	1.27 (0.45, 3.18)	1.80 (0.92, 3.37)	0.57 (0.31, 1.00)	**0.45 (0.22**,** 0.84)**	**0.39 (0.15**,** 0.91)**
rrTNBC	**6.12 (2.80**,** 13.4)**	**7.72 (4.34**,** 13.7)**	**2.34 (1.39**,** 3.86)**	1.17 (0.60, 2.14)	0.94 (0.45, 1.98)

Abbreviations: ACT, Adjuvant chemotherapy; ART, Adjuvant radiotherapy; BCS, Breast-conserving surgery; CI, Confidence interval; lrTNBC, Late relapse triple-negative breast cancer; OR, Odds ratio; rrTNBC, Rapid relapse triple-negative breast cancer. Logistic regression models were adjusted for relapse or death group, post-operative tumor size, tumor grade, and axillary lymph node status. Statistically significant models are shown in bold

A majority (*n* = 6926; 84%) of patients were born in Sweden, particularly in the lrTNBC group (Table 4). Notable differences in parental background were also observed: significantly more patients in the event-free group had parents born in Sweden (73% for fathers and 75% for mothers) than those in the rrTNBC (53% for both fathers and mothers) and lrTNBC (54% for fathers and 57% for mothers) groups. Furthermore, rrTNBC patients were less likely to be married, employed, and have pursued higher education, but more likely to have lower household incomes (Q1 and Q2).

**Table 4:** Sociodemographic features of triple-negative breast cancer patients (*n* = 8298), stratified by time to relapse or death

Characteristic	Overall (*n* = 8298)	Rapid relapse or death (*n* = 1236)	Late relapse or death (*n* = 1506)	Event-free at last follow-up (*n* = 5556)	*P*
**Patient’s birth country (%)**					**< 0.001**
Sweden	6926 (83.5)	1006 (81.4)	1293 (85.9)	4627 (83.3)	
Africa	67 (0.8)	9 (0.7)	12 (0.8)	46 (0.8)	
Asia	305 (3.7)	37 (3.0)	31 (2.1)	237 (4.3)	
EU28, non-Nordic countries	315 (3.8)	54 (4.4)	50 (3.3)	211 (3.8)	
Europe, non-EU28 and Nordic countries	177 (2.1)	27 (2.2)	22 (1.5)	128 (2.3)	
North America	22 (0.3)	6 (0.5)	2 (0.1)	14 (0.3)	
Nordic countries, not Sweden	404 (4.9)	77 (6.2)	81 (5.4)	246 (4.4)	
Oceania	3 (0.0)	0 (0.0)	0 (0.0)	3 (0.1)	
Former Soviet Union	5 (0.1)	1 (0.1)	0 (0.0)	4 (0.1)	
South America	45 (0.5)	6 (0.5)	4 (0.3)	35 (0.6)	
Missing data	29 (0.3)	13 (1.1)	11 (0.7)	5 (0.1)	
**Father’s birth country (%)**					**< 0.001**
Sweden	5512 (66.4)	652 (52.8)	808 (53.7)	4052 (72.9)	
Africa	7 (0.1)	0 (0.0)	1 (0.1)	6 (0.1)	
Asia	23 (0.3)	2 (0.2)	3 (0.2)	18 (0.3)	
EU28, non-Nordic countries	122 (1.5)	11 (0.9)	15 (1.0)	96 (1.7)	
Europe, non-EU28 and Nordic countries	46 (0.6)	8 (0.6)	3 (0.2)	35 (0.6)	
North America	9 (0.1)	2 (0.2)	0 (0.0)	7 (0.1)	
Nordic countries, not Sweden	227 (2.7)	24 (1.9)	40 (2.7)	163 (2.9)	
Former Soviet Union	5 (0.1)	1 (0.1)	0 (0.0)	4 (0.1)	
South America	10 (0.1)	0 (0.0)	2 (0.1)	8 (0.1)	
Missing data	2337 (28.2)	536 (43.4)	634 (42.1)	1167 (21.0)	
**Mother’s birth country (%)**					**< 0.001**
Sweden	5666 (68.3)	655 (53.0)	851 (56.5)	4160 (74.9)	
Africa	4 (0.0)	0 (0.0)	1 (0.1)	3 (0.1)	
Asia	30 (0.4)	1 (0.1)	5 (0.3)	24 (0.4)	
EU28, non-Nordic countries	121 (1.5)	18 (1.5)	13 (0.9)	90 (1.6)	
Europe, non-EU28 and Nordic countries	49 (0.6)	8 (0.6)	3 (0.2)	38 (0.7)	
North America	12 (0.1)	1 (0.1)	2 (0.1)	9 (0.2)	
Nordic countries, not Sweden	319 (3.8)	43 (3.5)	54 (3.6)	222 (4.0)	
Former Soviet Union	4 (0.0)	0 (0.0)	0 (0.0)	4 (0.1)	
South America	16 (0.2)	2 (0.2)	1 (0.1)	13 (0.2)	
Missing data	2077 (25.0)	508 (41.1)	576 (38.2)	993 (17.9)	
**Marital status (%)**					**< 0.001**
Single	1508 (18.2)	172 (13.9)	220 (14.6)	1116 (20.1)	
Married	3993 (48.1)	429 (34.7)	642 (42.6)	2922 (52.6)	
Registered partner	3 (0.0)	0 (0.0)	2 (0.1)	1 (0.0)	
Divorced	1521 (18.3)	193 (15.6)	265 (17.6)	1063 (19.1)	
Widowed	1098 (13.2)	288 (23.3)	366 (24.3)	444 (8.0)	
Missing data	175 (2.1)	154 (12.5)	11 (0.7)	10 (0.2)	
**Household income (%)**					**< 0.001**
Q1	2102 (25.3)	415 (33.6)	577 (38.3)	1110 (20.0)	
Q2	2002 (24.1)	289 (23.4)	367 (24.4)	1346 (24.2)	
Q3	1996 (24.1)	205 (16.6)	276 (18.3)	1515 (27.3)	
Q4	2023 (24.4)	173 (14.0)	275 (18.3)	1575 (28.3)	
Missing data	175 (2.1)	154 (12.5)	11 (0.7)	10 (0.2)	
**Educational level (%)**					**< 0.001**
< High school	1716 (20.7)	341 (27.6)	479 (31.8)	896 (16.1)	
High school	3429 (41.3)	406 (32.8)	594 (39.4)	2429 (43.7)	
Higher education	2916 (35.1)	319 (25.8)	411 (27.3)	2186 (39.3)	
Missing data	237 (2.9)	170 (13.8)	22 (1.5)	45 (0.8)	
**Employment (%)**					**< 0.001**
Employed	3899 (47.0)	341 (27.6)	431 (28.6)	3127 (56.3)	
Unemployed	4211 (50.7)	739 (59.8)	1064 (70.7)	2408 (43.3)	
Missing data	188 (2.3)	156 (12.6)	11 (0.7)	21 (0.4)	

Abbreviations: EU28, European Union with all 28 member states; Q, Quartile

P-values were calculated using Chi-square test for categorical variables (with continuity correction) and ANOVA for continuous variables

Overall, logistic regression showed that rrTNBC was associated with adverse tumor characteristics, including larger tumor size and axillary lymph node involvement (Table 5). These associations were robust and showed large effect sizes, indicating an increased likelihood of rapid distant relapse or death among patients presenting with advanced disease at diagnosis. These patients were also more likely to be older, have mothers born in non-Nordic EU28 countries, and be single or widowed, while patients with a high school education were less likely to belong to the rrTNBC group. Patients with lrTNBC were more likely to present with early-stage disease and be unemployed, but were less likely to belong to the Q2/Q3 household income quartiles. Finally, patients in the event-free group were more likely to be younger, have small and low-grade tumors, be pN0, have higher household incomes, be highly educated, and employed.

**Table 5:** Multivariable logistic regression analysis showing factors associated with rapid relapse (rrTNBC), late relapse (lrTNBC), and event-free status

	OR (95% CI)		
	rrTNBC	lrTNBC	non-rrTNBC
**Patient age**	**1.02 (1.00**,** 1.03)**	1.01 (1.00, 1.02)	**0.99 (0.98**,** 0.99)**
**Tumor size**	**1.03 (1.03**,** 1.04)**	**1.01 (1.01**,** 1.02)**	**0.97 (0.97**,** 0.97)**
**NHG grade**			
NHG grade 1	1.00	1.00	1.00
NHG grade 2	1.63 (0.56, 4.69)	1.70 (0.88, 3.27)	0.56 (0.31, 1.01)
NHG grade 3	2.62 (0.94, 7.33)	1.56 (0.82, 2.95)	**0.50 (0.28**,** 0.89)**
**pN**			
pN0	1.00	1.00	1.00
pN1	**2.82 (2.23**,** 3.56)**	1.10 (0.88, 1.38)	**0.53 (0.44**,** 0.64)**
pN2	**3.43 (1.04**,** 11.33)**	1.38 (0.43, 4.36)	**0.35 (0.13**,** 0.95)**
pN3	**3.47 (1.03**,** 11.64)**	2.08 (0.68, 6.33)	**0.25 (0.08**,** 0.77)**
pNX	2.11 (0.69, 6.42)	1.37 (0.56, 3.33)	0.49 (0.22, 1.10)
**Birth country**			
Sweden	1.00	1.00	1.00
EU28, not Nordic	0.24 (0.03, 2.08)	0.51 (0.09, 2.97)	2.92 (0.71, 12.02)
Europe, not EU28 or Nordic	2.20 (0.24, 19.85)	0.65 (0.05, 7.78)	0.95 (0.17, 5.21)
North America	4.32 (0.41, 45.91)	1.83E.06 (0, Inf)	0.68 (0.07, 6.59)
Nordic, not Sweden	0.62 (0.20, 1.95)	2.19 (1.00, 4.78)	0.64 (0.31, 1.31)
**Father’s birth country**			
Sweden	1.00	1.00	1.00
EU28, not Nordic	0.71 (0.27, 1.86)	1.12 (0.54, 2.33)	1.04 (0.55, 1.95)
Europe, not EU28 or Nordic	1.48 (0.24, 9.17)	0.30 (0.03, 3.03)	1.31 (0.28, 6.08)
North America	3.12 (0.58, 16.83)	1.17E-06 (0, Inf)	0.96 (0.18, 5.00)
Nordic, not Sweden	0.90 (0.44, 1.85)	0.97 (0.55, 1.71)	1.07 (0.66, 1.74)
**Mother’s birth country**			
Sweden	1.00	1.00	1.00
EU28, not Nordic	**2.62 (1.15**,** 5.95)**	0.68 (0.27, 1.71)	0.72 (0.37, 1.42)
Europe, not EU28 or Nordic	0.64 (0.07, 6.11)	2.12 (0.32, 13.96)	0.91 (0.19, 4.46)
North America	0.22 (0.013, 3.74)	0.80 (0.15, 4.09)	1.64 (0.33, 8.04)
Nordic, not Sweden	1.43 (0.83, 2.47)	1.11 (0.70, 1.74)	0.79 (0.54, 1.16)
**Marital status**			
Married	1.00	1.00	1.00
Divorced	1.01 (0.75, 1.36)	0.94 (0.74, 1.19)	1.04 (0.85, 1.28)
Single	**1.36 (1.01**,** 1.82)**	1.05 (0.83, 1.33)	0.83 (0.68, 1.02)
Widowed	**1.45 (1.02**,** 2.06)**	1.14 (0.85, 1.53)	**0.74 (0.57**,** 0.95)**
**Household income**			
Q1	1.00	1.00	1.00
Q2	0.98 (0.73, 1.32)	**0.74 (0.58**,** 0.93)**	**1.29 (1.04**,** 1.59)**
Q3	0.78 (0.56, 1.09)	**0.71 (0.55**,** 0.92)**	**1.45 (1.16**,** 1.82)**
Q4	0.82 (0.56, 1.17)	0.78 (0.59, 1.03)	**1.31 (1.03**,** 1.66)**
**Educational level**			
< High school	1.00	1.00	1.00
High school	**0.75 (0.57**,** 0.98)**	0.81 (0.65, 1.01)	**1.37 (1.13**,** 1.66)**
Higher education	0.84 (0.62, 1.13)	0.83 (0.65, 1.05)	**1.28 (1.04**,** 1.57)**
**Employment**			
Employed	1.00	1.00	1.00
Unemployed	1.23 (0.92, 1.66)	**1.50 (1.20**,** 1.89)**	**0.69 (0.57**,** 0.84)**

Abbreviations: CI, Confidence interval; lrTNBC, Late relapse triple-negative breast cancer; OR, Odds ratio; Q, Quartile; rrTNBC, Rapid relapse triple-negative breast cancer. Logistic regression models were adjusted for all covariates in the table. Statistically significant models are shown in bold

### rrTNBC consistently associated with worse clinical outcomes

**Fig. 3:**
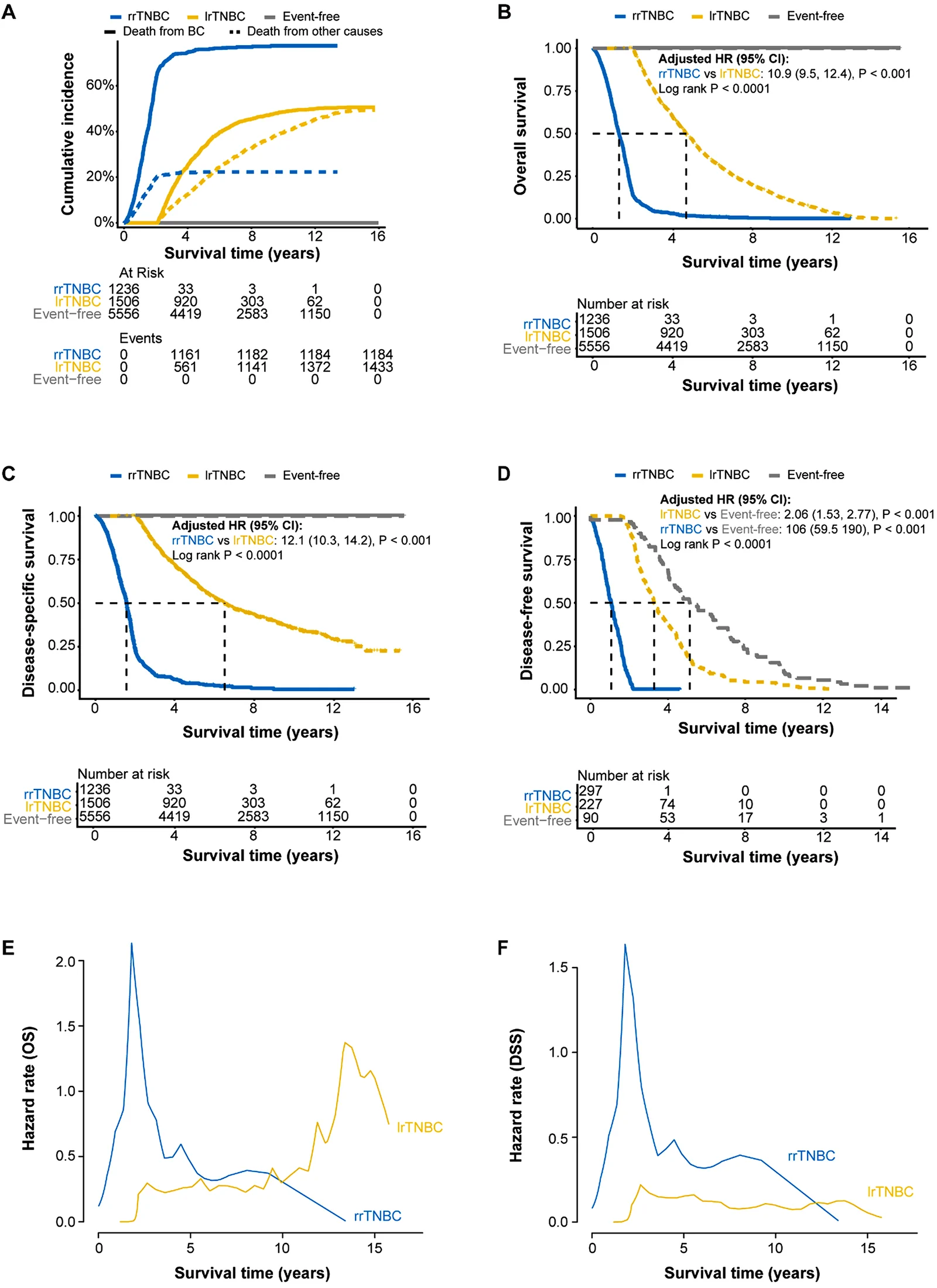
Survival outcomes stratified by time to relapse or death in triple-negative breast cancer (TNBC; *n* = 8298). (**A**) Competing risk survival analysis of death from breast cancer (BC; solid line) or other causes (dotted line) in TNBC patients stratified by time to relapse or death (rrTNBC: rapid relapse or death; lrTNBC: late relapse or death; event-free at last follow-up). Kaplan–Meier curves compare (**B**) overall survival (OS), (**C**) disease-specific survival (DSS), and (**D**) disease-free survival among patients with rrTNBC, lrTNBC, and event-free disease. Smoothed group-specific hazard functions for (**E**) OS and (**F**) DSS in patients with rrTNBC and lrTNBC. The Event-free group was excluded due to the lack of events. Multivariable Cox regression models were adjusted for time to relapse or death, patient age, tumor size, Nottingham histological grade (NHG), axillary lymph node status, CCIw group, and adjuvant chemotherapy received. Adjusted hazard ratios (HRs) with 95% confidence intervals (95% CI) and log-rank P-values are shown, with numbers at risk displayed below each panel. The x-axes depict years after initial diagnosis and the y-axes depict survival probabilities

Given the differences in time to relapse or death, we then performed survival analysis. Patients with rrTNBC had significantly worse outcomes than those with lrTNBC or event-free patients across all clinical endpoints: cumulative incidence, overall survival (OS), disease-specific survival (DSS), and disease-free survival (DFS; Fig. 3). rrTNBC was characterized by early and high breast cancer-related mortality, resulting in the poorest OS and DSS probabilities. lrTNBC was associated with intermediate outcomes, with gradual recurrences and death over time. Event-free patients consistently had the most favorable prognoses. Multivariate Cox regression analysis showed that patients with rrTNBC had an approximately 10- (OS) and 12-fold (DSS) higher risk of death compared to patients with lrTNBC, whereas patients with lrTNBC and rrTNBC had an approximately 2- and 100-fold higher risk of recurrence (DFS) than event-free patients, respectively. These findings were consistent in the sensitivity analyses restricted to regions with complete relapse data (Supplementary Figure S1 and Supplementary Tables S5-S7).

## Discussion

In this nationwide population-based cohort of more than 8000 patients with TNBC, we demonstrate that rapid distant relapse or death within 24 months identifies a clinically distinct and highly vulnerable subgroup characterized by aggressive tumor features, early multi-organ metastatic spread, inferior survival outcomes, and sociodemographic disparities. In contrast, late relapse TNBC represents an intermediate phenotype, whereas event-free patients display a more favorable biological and socioeconomic profile. Our findings that rrTNBC patients present with larger primary tumors, more advanced nodal involvement, higher pathological stage, and substantially shorter time to distant relapse are consistent with previous reports describing the pronounced early relapse pattern of TNBC. Several large cohort studies have shown that the median time to distant metastasis in TNBC is approximately two years, thereby highlighting the biological aggressiveness of this subtype [5, 10].

The clinical definition of rapid relapse with 24 months should be distinguished from treatment resistance, which may be defined more narrowly as disease recurrence or progression during or shortly after systemic therapy. Although the 24-month threshold is a categorical simplification, it was prespecified and is consistent with previous studies defining rapid-relapse TNBC according to distant relapse or death within two years after diagnosis [6, 11, 13, 19]. This definition describes an early clinical relapse phenotype and should not be equated with the narrower concept of treatment-resistant disease, such as recurrence during or shortly after adjuvant chemotherapy.

The significantly higher metastatic burden and broader organ involvement observed in rrTNBC compared with lrTNBC further support the concept that rrTNBC represents a biologically aggressive disease entity with early systemic dissemination. This aligns with prior observations that early TNBC relapses are frequently associated with visceral and central nervous system metastases and poor post-relapse survival [9, 30]. While most TNBC recurrences occur within the first few years after diagnosis, our data confirm that a substantial proportion of patients experience distant relapse beyond 24 months. The lrTNBC group exhibited intermediate tumor characteristics and survival outcomes, supporting prior reports that late relapse TNBC represents a biologically and clinically distinct subgroup rather than random late events [14]. The longer time to recurrence and lower metastatic burden in lrTNBC may reflect differences in tumor dormancy, immune surveillance, or host-tumor interactions, highlighting the heterogeneity of TNBC beyond its immunohistochemical definition [2, 30]. The consistently higher frequency of multi-organ and visceral metastases observed in rrTNBC may reflect early clonal dissemination and limited metastatic latency, in contrast to lrTNBC where delayed recurrence may involve tumor dormancy or immune-mediated restraint [2, 10, 30].

We observed that rrTNBC patients were less likely to receive ACT and ART and more likely to undergo mastectomy and axillary dissection. Although tumor stage partly explains these differences, prior studies have shown that age and higher comorbidity burden are independently associated with reduced likelihood of receiving guideline-concordant treatment in TNBC [20, 24]. Higher comorbidity burden, commonly assessed using the Charlson comorbidity index, has also been associated with inferior outcomes and reduced treatment tolerance in TNBC and metastatic breast cancer, potentially contributing to early disease progression and relapse [22]. Importantly, our age-stratified analyses demonstrated that treatment disparities persisted across age groups, with both rrTNBC and lrTNBC patients being less likely to receive BCS with ART, and older patients (≥ 50 years) being less likely to receive ACT. Furthermore, the likelihood of NACT declined after 65 years of age, particularly among patients with lrTNBC, suggesting that chronological age remains a key determinant of treatment intensity in TNBC [20, 23, 24]. Although rrTNBC patients were more likely to undergo mastectomy and less likely to receive ART, these treatment patterns are unlikely to represent causal drivers of rapid distant relapse. This likely reflects confounding, where aggressive tumor characteristics, advanced stage, older age, and comorbidity influence both surgical choice and the risk of early systemic progression [5, 9, 10, 30]. In contrast, reduced exposure to systemic therapy, particularly NACT or ACT, may contribute to inferior outcomes in some patients [4, 20, 22–24]. However, rrTNBC is generally considered to reflect early metastatic dissemination driven by tumor biology rather than undertreatment [2, 5, 9, 10, 30]. Nevertheless, our previous nationwide study showed that older patients had a greater comorbidity burden and were less likely to receive several treatment modalities. Patients with a high comorbidity burden also had larger tumors and more locoregionally advanced disease. Importantly, patients aged ≥ 75 years had a higher risk of breast cancer-related death during the first eight years of follow-up, whereas mortality from other causes predominated thereafter, indicating that their poorer prognosis cannot be attributed solely to competing mortality [20]. It might also be worth mentioning that mammography screening in Sweden ends at age 74. This may contribute to a higher stage at diagnosis and poorer prognosis in this age group.

Beyond tumor-intrinsic features, our study highlights sociodemographic differences across the relapse groups. Patients with rrTNBC were more likely to be older, single or widowed, unemployed, have lower household income, and parental country of birth. The observed association between maternal country of birth and rapid relapse should be interpreted with caution. Given the large number of sociodemographic variables and comparisons examined, this finding may represent a chance association due to multiple testing rather than a clinically meaningful relationship, thereby warranting further studies to determine whether this association is reproducible. These findings are in line with previous population-based studies demonstrating that individual-level socioeconomic factors, such as insurance status, income, and marital status, are associated with rapid relapse in TNBC, even after adjustment for tumor stage [11, 13]. These disparities may reflect differences in healthcare access, social support, comorbidity burden, and treatment completion, all of which can influence early disease progression in aggressive cancers such as TNBC. The observed associations suggest that sociodemographic differences in treatment patterns and outcomes may persist even within a tax-funded healthcare system with universal coverage. Universal coverage may reduce financial barriers but does not necessarily ensure equal access to, acceptance of, or completion of guideline-recommended care. These findings highlight the importance of systematically evaluating whether potentially modifiable barriers, including differences in social support, health literacy, care navigation, and engagement with cancer care, contribute to the observed patterns. However, performance status, frailty, treatment contraindications, patient preferences, and treatment completion were not available in the registers. The underlying mechanisms therefore could not be determined, and the observed associations should be regarded as hypothesis-generating, particularly in view of the multiple comparisons performed [11, 13, 19].

The survival differences observed between rrTNBC and lrTNBC highlight the clinical importance of distinguishing these relapse patterns. Patients with rrTNBC exhibited significantly worse OS, DSS, and DFS, with a 10-12-fold higher risk of death compared with lrTNBC, and an increased risk of recurrence relative to event-free patients. The sensitivity analysis restricted to patients with complete relapse reporting supported the main findings. However, when survival was calculated from the reported date of distant metastasis, the differences were attenuated: rrTNBC remained independently associated with higher disease-specific mortality, whereas the adjusted difference in overall mortality was not statistically significant. Failure to separate these groups may obscure clinically meaningful heterogeneity, dilute prognostic models, and limit the identification of patients at highest risk. This study is subject to limitations inherent to registry-based analyses, including missingness in relapse and chemotherapy variables, as well as potential confounding. Since relapse data were only available from four of the six Swedish healthcare regions at the time of data extraction and survival data were complete nationwide, patients from regions without relapse reporting contributed only to early mortality analyses, which may explain the lower representation of these regions among rrTNBC patients. In addition, classification as event-free was based on the absence of distant relapse or death by the end of the available follow-up. However, the large nationwide cohort, long follow-up, and consistent findings across multiple outcome measures and in the sensitivity analyses (restricted to healthcare regions with relapse data) support the robustness of the observed associations.

Future studies should focus on understanding the biological factors (e.g., TNBC subtyping, HER2-low status, and stromal-immune microenvironment) that contribute to relapse [10]. Investigating tumor heterogeneity, the evolution of resistant cells, and the tumor microenvironment will be crucial to developing solutions to relapse. Additionally, identifying biomarkers to predict relapse risk is vital for personalizing follow-up care and enabling early interventions [31]. Advances in molecular genetic profiling from patients have the potential to detect minimal residual disease (MRD) and circulating tumor DNA (ctDNA), which can act as early signs of relapse [32].Alongside the development of novel therapeutic strategies, efforts are needed to ensure equitable assessment and delivery of guideline-recommended care across age and sociodemographic groups. Future studies should examine whether the observed treatment patterns reflect differences in clinical eligibility, contraindications, patient preferences, treatment acceptance or completion, or potentially modifiable barriers within the healthcare system. Such studies may identify opportunities for more equitable care and improved outcomes [33, 34]. It is also crucial to consider the impact of relapse on patients’ quality of life and mental well-being, emphasizing the need for comprehensive survivorship programs that offer continuous monitoring and support. Early identification of patients at risk for rapid relapse could enable intensified surveillance, prioritization for clinical trials, or tailored treatment strategies, while improved characterization of late relapse TNBC may help avoid overtreatment and guide long-term follow-up strategies.

This study is reported in accordance with the STROBE (Strengthening the Reporting of Observational Studies in Epidemiology) guidelines.

## Supplementary Information

Below is the link to the electronic supplementary material.

Supplementary Material 1

## Data Availability

The data that support the findings of this study were obtained from the Swedish National Breast Cancer Register, the Swedish Patient Register, the Swedish Cause of Death Register, and Statistics Sweden. Restrictions apply to the availability of these data, which were used under ethical approval and data-sharing agreements for the current study and are therefore not publicly available. Access to the data may be granted upon reasonable request and with permission from the relevant Swedish authorities and ethical review boards.
