# Supplementary material for: Sociodemographic and clinicopathologic features associated with rapid relapse in triple-negative breast cancer: a Swedish nationwide registry-based study
Source: Breast Cancer Res Treat. 2026 Oct 28;220(1):19. doi: 10.1007/s10549-026-08115-z (PMC13719746; doi:10.1007/s10549-026-08115-z)
Supplement: Supplementary file 1 [file 10549_2026_8115_MOESM1_ESM.docx]

**Supplementary material**

**Sociodemographic and clinicopathologic features associated with rapid relapse in triple-negative breast cancer: A Swedish nationwide registry-based study**

Sithumini Sarathchandra, Jenny Nyqvist-Streng, Elin Grönberg, Zakaria Einbeigi, Khalil Helou, Anikó Kovács, Slavica Janeva, Per Karlsson, Toshima Z. Parris

Contents

[Figure S1	3](#_Toc240829489)

[Figure S2	4](#_Toc240829490)

[Table S1	6](#_Toc240829491)

[Table S2	7](#_Toc240829492)

[Table S3	9](#_Toc240829493)

[Table S4	10](#_Toc240829494)

[Table S5	12](#_Toc240829495)

[Table S6	14](#_Toc240829496)

[Table S7	15](#_Toc240829497)

# Figure S1

**Figure S1.** Survival outcomes stratified by time to relapse or death in triple-negative breast cancer (TNBC); sensitivity analysis (n = 5846) after removing 2452 patients diagnosed in healthcare regions lacking relapse data. (A) Competing risk survival analysis of death from breast cancer (BC; solid line) or other causes (dotted line) in TNBC patients stratified by time to relapse or death (rrTNBC: rapid relapse or death; lrTNBC: late relapse or death; event-free at last follow-up). Kaplan–Meier curves compare (B) overall survival, (C) disease-specific survival, and (D) disease-free survival among patients with rrTNBC, lrTNBC, and event-free disease. Multivariable Cox regression models were adjusted for time to relapse or death, patient age, tumor size, Nottingham histological grade (NHG), axillary lymph node status, CCIw group, and adjuvant chemotherapy received. Adjusted hazard ratios (HRs) with 95% confidence intervals (95% CI) and log-rank P-values are shown, with numbers at risk displayed below each panel. The x-axes depict years after initial diagnosis and the y-axes depict survival probabilities.

# Figure S2

**Figure S2.** Survival outcomes stratified by time to relapse or death in 704 patients with triple-negative breast cancer (TNBC) developing distant metastases (with a reported date of metastasis; sensitivity analysis). (A) Competing risk survival analysis of death from breast cancer (BC; solid line) or other causes (dotted line) in TNBC patients stratified by time to relapse or death (rrTNBC: rapid relapse or death; lrTNBC: late relapse or death). Kaplan–Meier curves compare (B) overall survival (time from the date of distant metastasis to death of any cause) and (C) disease-specific survival (time from the date of distant metastasis to breast cancer-related death) among patients with rrTNBC and lrTNBC disease. Multivariable Cox regression models were adjusted for time to relapse or death, patient age, tumor size, Nottingham histological grade (NHG), axillary lymph node status, CCIw group, and adjuvant chemotherapy received. Adjusted hazard ratios (HRs) with 95% confidence intervals (95% CI) and log-rank P-values are shown, with numbers at risk displayed below each panel. The x-axes depict days after reported distant metastasis and the y-axes depict survival probabilities.

# Table S1

| **Percentages for 1-, 3-, and 5-year survival probabilities for overall survival, disease-specific survival, and disease-free survival, stratified by relapse or death group** | | | | | | | | | | | |
| --- | --- | --- | --- | --- | --- | --- | --- | --- | --- | --- | --- |
|  | **1 year (95% CI)** | | |  | **3 year (95% CI)** | | |  | **5 year (95% CI)** | | |
| **Age groups** | **rrTNBC** | **lrTNBC** | **Event-free** |  | **rrTNBC** | **lrTNBC** | **Event-free** |  | **rrTNBC** | **lrTNBC** | **Event-free** |
| **OS** |  |  |  |  |  |  |  |  |  |  |  |
| All patients | 68 (65.1, 70.3) | 100 (100, 100) | 100 (100, 100) |  | 6 (4.4, 7.1) | 79 (76.8, 80.9) | 100 (100, 100) |  | 2 (1.1, 2.9) | 48 (45.5, 50.6) | 100 (100, 100) |
| <40 years | 83 (75.5, 90.8) | 100 (100, 100) | 100 (100, 100) |  | 7 (3.1, 16.1) | 75 (66.2, 84.8) | 100 (100, 100) |  | 4 (1.4, 12.6) | 37 (28.0, 49.2) | 100 (100, 100) |
| 40-49 years | 83 (76.5, 89.0) | 100 (100, 100) | 100 (100, 100) |  | 10 (6.2, 17.2) | 80 (73.5, 86.8) | 100 (100, 100) |  | 5 (2.3, 11.2) | 47 (39.4, 56.2) | 100 (100, 100) |
| 50-64 years | 76 (71.1, 82.1) | 100 (100, 100) | 100 (100, 100) |  | 7 (4.2, 11.2) | 81 (76.5, 84.7) | 100 (100, 100) |  | 2 (0.7, 5.4) | 47 (42.3, 52.8) | 100 (100, 100) |
| 65-74 years | 67 (61.4, 73.8) | 100 (100, 100) | 100 (100, 100) |  | 7 (4.7, 12.0) | 81 (76.8, 85.1) | 100 (100, 100) |  | 2 (0.9, 5.8) | 56 (50.5, 61.0) | 100 (100, 100) |
| ≥75 years | 58 (53.6, 61.9) | 100 (100, 100) | 100 (100, 100) |  | 3 (1.6, 4.6) | 77 (73.3, 80.3) | 100 (100, 100) |  | 0.4 (0.1, 1.7) | 46 (41.7, 49.9) | 100 (100, 100) |
| **DSS** |  |  |  |  |  |  |  |  |  |  |  |
| All patients | 75 (72.5, 77.5) | 100 (100, 100) | 100 (100, 100) |  | 10 (7.9, 12.2) | 85 (83.5, 87.2) | 100 (100, 100) |  | 4 (2.7, 6.1) | 63 (60.4, 65.7) | 100 (100, 100) |
| <40 years | 86 (78.9, 93.2) | 100 (100, 100) | 100 (100, 100) |  | 10 (4.7, 21.6) | 76 (67.5, 85.8) | 100 (100, 100) |  | 8 (2.9, 19.5) | 39 (29.3, 50.9) | 100 (100, 100) |
| 40-49 years | 84 (78.7, 90.6) | 100 (100, 100) | 100 (100, 100) |  | 14 (9.1, 22.4) | 83 (77.1, 89.7) | 100 (100, 100) |  | 8 (3.8, 16.0) | 55 (47.0, 64.5) | 100 (100, 100) |
| 50-64 years | 80 (74.8, 85.2) | 100 (100, 100) | 100 (100, 100) |  | 9 (5.7, 14.7) | 84 (79.8, 87.5) | 100 (100, 100) |  | 3 (1.0, 7.8) | 56 (51.0, 61.8) | 100 (100, 100) |
| 65-74 years | 75 (69.6, 81.4) | 100 (100, 100) | 100 (100, 100) |  | 12 (7.9, 18.8) | 86 (82.5, 89.9) | 100 (100, 100) |  | 5 (2.1, 10.3) | 68 (63.4, 73.7) | 100 (100, 100) |
| ≥75 years | 68 (64.0, 72.2) | 100 (100, 100) | 100 (100, 100) |  | 7 (4.5, 11.5) | 88 (85.2, 90.8) | 100 (100, 100) |  | 2 (0.8, 6.4) | 71 (66.8, 75.2) | 100 (100, 100) |
| **DFS** |  |  |  |  |  |  |  |  |  |  |  |
| All patients | 49 (43.5, 54.9) | 100 (100, 100) | 98 (94.8, 100) |  | 0.3 (0.05, 2.4) | 50 (44.1, 57.2) | 82 (74.7, 90.5) |  | - | 14 (9.9, 18.9) | 49 (39.6, 60.4) |
| <40 years | 41 (28.2, 59.8) | 100 (100, 100) | 92 (78.9, 100) |  | - | 29 (14.5, 56.2) | 62 (40.0, 94.6) |  | - | 10 (2.6, 35.6) | 46 (25.7, 83.0) |
| 40-49 years | 48 (36.0, 64.1) | 100 (100, 100) | 100 (100, 100) |  | - | 63 (48.7, 81.1) | 88 (72.7, 100) |  | - | 14 (6.4, 32.2) | 56 (36.5, 86.7) |
| 50-64 years | 44 (33.1, 57.8) | 100 (100, 100) | 96 (89.8, 100) |  | 2 (0.2, 10.9) | 49 (39.1, 62.2) | 89 (78.5, 100) |  | - | 18 (10.9, 29.2) | 46 (31.2, 69.1) |
| 65-74 years | 59 (47.4, 73.3) | 100 (100, 100) | 100 (100, 100) |  | - | 46 (34.1, 62.1) | 82 (67.2, 99.6) |  | - | 12 (5.7, 25.4) | 46 (28.8, 71.8) |
| ≥75 years | 50 (40.6, 61.6) | 100 (100, 100) | 100 (100, 100) |  | - | 56 (43.8, 72.2) | 82 (61.9, 100) |  | - | 10 (4.5, 23.9) | 55 (31.8, 93.6) |

Abbreviations: CI, Confidence interval; DFS, disease-free survival; DSS, disease-specific survival; lrTNBC, late relapse triple-negative breast cancer; OS, overall survival; rrTNBC, rapid relapse triple-negative breast cancer

# Table S2

| **Patient and tumor characteristics of triple-negative breast cancer patients with CNS metastases (n = 128), stratified by time to relapse or death** | | | | |
| --- | --- | --- | --- | --- |
| **Characteristic** | **Overall (n = 128)** | **Rapid relapse or death (n = 75)** | **Late relapse or death (n = 53)** | **P** |
| **Swedish region (%)** |  |  |  | **0.014** |
| Stockholm/Gotland | 64 (50.0) | 31 (41.3) | 33 (62.3) |  |
| South | 27 (21.1) | 23 (30.7) | 4 (7.5) |  |
| Southeast | 14 (10.9) | 8 (10.7) | 6 (11.3) |  |
| West | 23 (18.0) | 13 (17.3) | 10 (18.9) |  |
| **Age, years, median [IQR]** | 56.5 [44.0, 65.0] | 57.0 [45.0, 66.5] | 53.0 [42.0, 62.0] | 0.139 |
| **Age group, years (%)** |  |  |  | **0.047** |
| <40 | 19 (14.8) | 7 (9.3) | 12 (22.6) |  |
| 40-49 | 31 (24.2) | 20 (26.7) | 11 (20.8) |  |
| 50-64 | 43 (33.6) | 25 (33.3) | 18 (34.0) |  |
| 65-74 | 20 (15.6) | 10 (13.3) | 10 (18.9) |  |
| ≥75 | 15 (11.7) | 13 (17.3) | 2 (3.8) |  |
| **Clinical stage (%)** |  |  |  | **0.005** |
| IA | 24 (18.8) | 8 (10.7) | 16 (30.2) |  |
| IIA | 45 (35.2) | 24 (32.0) | 21 (39.6) |  |
| IIB | 41 (32.0) | 29 (38.7) | 12 (22.6) |  |
| IIIA | 11 (8.6) | 10 (13.3) | 1 (1.9) |  |
| IIIB | 3 (2.3) | 3 (4.0) | 0 (0.0) |  |
| IIIC | 3 (2.3) | 1 (1.3) | 2 (3.8) |  |
| Unspecified | 1 (0.8) | 0 (0.0) | 1 (1.9) |  |
| **Tumor size, mm, median [IQR]*** | 39.0 [28.8, 52.5] | 36.0 [27.5, 55.0] | 42.0 [42.0, 42.0] | 0.827 |
| **Ki67% (mean (SD))*** | 61.3 (23.3) | 66.2 (23.7) | 54.3 (21.3) | **0.04** |
| **Pathological stage (%)†** |  |  |  | 0.352 |
| 0 | 1 (0.8) | 1 (1.3) | 0 (0.0) |  |
| IA | 22 (17.2) | 8 (10.7) | 14 (26.4) |  |
| IIA | 38 (29.7) | 22 (29.3) | 16 (30.2) |  |
| IIB | 14 (10.9) | 9 (12.0) | 5 (9.4) |  |
| IIIA | 19 (14.8) | 13 (17.3) | 6 (11.3) |  |
| IIIC | 11 (8.6) | 7 (9.3) | 4 (7.5) |  |
| Missing data | 23 (18.0) | 15 (20.0) | 8 (15.1) |  |
| **Cancer type (%)†** |  |  |  | 0.578 |
| TNBC-NST | 66 (51.6) | 40 (53.3) | 26 (49.1) |  |
| Other invasive cancer | 5 (3.9) | 3 (4.0) | 2 (3.8) |  |
| TNBC-ILC | 4 (3.1) | 1 (1.3) | 3 (5.7) |  |
| Missing data | 53 (41.4) | 31 (41.3) | 22 (41.5) |  |
| **Tumor size, mm, median [IQR]†** | 20.0 [15.0, 32.0] | 23.5 [15.8, 39.3] | 18.0 [12.0, 25.0] | **0.007** |
| **NHG (%)†** |  |  |  | 0.453 |
| Grade 1 | 0 (0.0) | 0 (0.0) | 0 (0.0) |  |
| Grade 2 | 11 (8.6) | 5 (6.7) | 6 (11.3) |  |
| Grade 3 | 64 (50.0) | 36 (48.0) | 28 (52.8) |  |
| **Ki67% (mean (SD))†** | 60.9 (29.4) | 66.0 (28.8) | 53.9 (29.2) | 0.106 |
| **Overall follow-up time, years (mean (SD))** | 3.0 (1.9) | 1.9 (1.2) | 4.5 (1.7) | **<0.001** |
| **Time to distant relapse or death, years (mean (SD))** | 2.1 (1.6) | 1.1 (0.5) | 3.5 (1.5) | **<0.001** |
| **Number of distant metastatic sites (%)** |  |  |  | 0.169 |
| 1 | 66 (51.6) | 43 (57.3) | 23 (43.4) |  |
| ≥2 | 62 (48.4) | 32 (42.7) | 30 (56.6) |  |
| **Comorbidities (%)** |  |  |  |  |
| Myocardial infarction | 2 (1.6) | 1 (1.3) | 1 (1.9) | 1 |
| Congestive heart failure | 0 (0.0) | 0 (0.0) | 0 (0.0) | **-** |
| Peripheral vascular disease | 2 (1.6) | 2 (2.7) | 0 (0.0) | 0.635 |
| Cerebrovascular disease | 6 (4.7) | 4 (5.3) | 2 (3.8) | 1 |
| Chronic obstructive pulmonary disease | 1 (0.8) | 1 (1.3) | 0 (0.0) | 1 |
| Chronic other pulmonary disease | 1 (0.8) | 0 (0.0) | 1 (1.9) | 0.861 |
| Rheumatic disease | 3 (2.3) | 2 (2.7) | 1 (1.9) | 1 |
| Dementia | 0 (0.0) | 0 (0.0) | 0 (0.0) | - |
| Hemiplegia | 1 (0.8) | 1 (1.3) | 0 (0.0) | 1 |
| Diabetes without chronic complication | 0 (0.0) | 0 (0.0) | 0 (0.0) | - |
| Diabetes with chronic complication | 0 (0.0) | 0 (0.0) | 0 (0.0) | - |
| Renal disease | 2 (1.6) | 2 (2.7) | 0 (0.0) | 0.635 |
| Mild liver disease | 1 (0.8) | 0 (0.0) | 1 (1.9) | 0.861 |
| Liver special | 0 (0.0) | 0 (0.0) | 0 (0.0) | - |
| Severe liver disease | 0 (0.0) | 0 (0.0) | 0 (0.0) | - |
| Peptic ulcer disease | 1 (0.8) | 1 (1.3) | 0 (0.0) | 1 |
| Malignancy | 14 (10.9) | 10 (13.3) | 4 (7.5) | 0.456 |
| Metastatic solid tumor | 0 (0.0) | 0 (0.0) | 0 (0.0) | - |
| AIDS | 0 (0.0) | 0 (0.0) | 0 (0.0) | - |
| **CCIw group (%)** |  |  |  | 0.282 |
| CCIw=0 | 99 (77.3) | 55 (73.3) | 44 (83.0) |  |
| CCIw=1-3 | 29 (22.7) | 20 (26.7) | 9 (17.0) |  |
| CCIw=4-10 | 0 (0.0) | 0 (0.0) | 0 (0.0) |  |

Abbreviations: CCIw, weighted Charlson comorbidity index; CNS, central nervous system; TNBC, Triple-negative breast cancer; TNBC–ILC, Triple-negative breast cancer–invasive lobular cancer; TNBC–NST, Triple-negative breast cancer of no special type. Statistically significant differences (P < 0.05) are highlighted in bold. P-values were calculated using Chi-square test for categorical variables (with continuity correction) and ANOVA for continuous variables.

# Table S3

| **Treatment characteristics of triple-negative breast cancer patients with CNS metastases (n = 128), stratified by time to relapse or death** | | | | |
| --- | --- | --- | --- | --- |
| **Characteristic** | **Overall (n = 128)** | **Rapid relapse or death (n = 75)** | **Late relapse or death (n = 53)** | **P** |
| **NACT type (%)** |  |  |  | 0.567 |
| Anthracycline-based | 21 (16.4) | 13 (17.3) | 8 (15.1) |  |
| Anthracycline+taxane | 48 (37.5) | 29 (38.7) | 19 (35.8) |  |
| Taxane-based | 4 (3.1) | 1 (1.3) | 3 (5.7) |  |
| Missing data | 55 (43.0) | 32 (42.7) | 23 (43.4) |  |
| **Axillary surgery type (%)** |  |  |  | 0.232 |
| SLNB | 39 (30.5) | 19 (25.3) | 20 (37.7) |  |
| AD | 62 (48.4) | 42 (56.0) | 20 (37.7) |  |
| Sampling (including SLNB) | 1 (0.8) | 0 (0.0) | 1 (1.9) |  |
| SLNB + AD | 23 (18.0) | 12 (16.0) | 11 (20.8) |  |
| **Surgery type (%)** |  |  |  | 0.562 |
| Breast-conserving surgery | 48 (37.5) | 25 (33.3) | 23 (43.4) |  |
| Mastectomy | 77 (60.2) | 48 (64.0) | 29 (54.7) |  |
| Subcutaneous mastectomy | 2 (1.6) | 1 (1.3) | 1 (1.9) |  |
| Missing data | 1 (0.8) | 1 (1.3) | 0 (0.0) |  |
| **Locoregional treatment (%)** |  |  |  | 0.898 |
| BCS+ART | 32 (25.0) | 19 (25.3) | 13 (24.5) |  |
| Mx-ART | 23 (18.0) | 12 (16.0) | 11 (20.8) |  |
| Mx+ART | 47 (36.7) | 29 (38.7) | 18 (34.0) |  |
| Other | 26 (20.3) | 15 (20.0) | 11 (20.8) |  |
| **ACT type (%)** |  |  |  | 0.448 |
| No ACT | 50 (39.1) | 32 (42.7) | 18 (34.0) |  |
| Anthracycline-based | 12 (9.4) | 4 (5.3) | 8 (15.1) |  |
| Anthracycline+taxane | 18 (14.1) | 11 (14.7) | 7 (13.2) |  |
| Taxane-based | 5 (3.9) | 3 (4.0) | 2 (3.8) |  |
| Other | 25 (19.5) | 13 (17.3) | 12 (22.6) |  |
| Missing data | 18 (14.1) | 12 (16.0) | 6 (11.3) |  |
| **ART (%)** | 81 (63.3) | 49 (65.3) | 32 (60.4) | 0.391 |
| **Adjuvant bisphosphonate therapy (%)** | 15 (11.7) | 5 (6.7) | 10 (18.9) | 0.098 |

Abbreviations: ACT, adjuvant chemotherapy; AD, axillary dissection; ART, adjuvant radiotherapy; BCS, breast-conserving surgery; CNS, central nervous system; Mx, mastectomy; NACT, neoadjuvant chemotherapy; SLNB, sentinel lymph-node biopsy. Statistically significant differences (P < 0.05) are highlighted in bold. P-values were calculated using Chi-square test for categorical variables (with continuity correction) and ANOVA for continuous variables.

# Table S4

| **Sociodemographic features of triple-negative breast cancer patients with CNS metastases (n = 128), stratified by time to relapse or death** | | | | |
| --- | --- | --- | --- | --- |
| **Characteristic** | **Overall (n = 128)** | **Rapid relapse or death (n = 75)** | **Late relapse or death (n = 53)** | **P** |
| **Patient's birth country (%)** |  |  |  | 0.443 |
| Sweden | 99 (77.3) | 56 (74.7) | 43 (81.1) |  |
| Asia | 8 (6.2) | 6 (8.0) | 2 (3.8) |  |
| EU28, non-Nordic countries | 7 (5.5) | 3 (4.0) | 4 (7.5) |  |
| Europe, non-EU28 and Nordic countries | 7 (5.5) | 6 (8.0) | 1 (1.9) |  |
| North America | 1 (0.8) | 1 (1.3) | 0 (0.0) |  |
| Nordic countries, not Sweden | 6 (4.7) | 3 (4.0) | 3 (5.7) |  |
| **Father's birth country (%)** |  |  |  | 0.647 |
| Sweden | 83 (64.8) | 48 (64.0) | 35 (66.0) |  |
| Asia | 2 (1.6) | 1 (1.3) | 1 (1.9) |  |
| EU28, non-Nordic countries | 5 (3.9) | 2 (2.7) | 3 (5.7) |  |
| Europe, non-EU28 and Nordic countries | 2 (1.6) | 2 (2.7) | 0 (0.0) |  |
| Nordic countries, not Sweden | 3 (2.3) | 1 (1.3) | 2 (3.8) |  |
| Missing data | 33 (25.8) | 21 (28.0) | 12 (22.6) |  |
| **Mother's birth country (%)** |  |  |  | 0.722 |
| Sweden | 80 (62.5) | 43 (57.3) | 37 (69.8) |  |
| Asia | 2 (1.6) | 1 (1.3) | 1 (1.9) |  |
| EU28, non-Nordic countries | 5 (3.9) | 3 (4.0) | 2 (3.8) |  |
| Europe, non-EU28 and Nordic countries | 1 (0.8) | 1 (1.3) | 0 (0.0) |  |
| Nordic countries, not Sweden | 9 (7.0) | 6 (8.0) | 3 (5.7) |  |
| Missing data | 31 (24.2) | 21 (28.0) | 10 (18.9) |  |
| **Marital status (%)** |  |  |  | 0.253 |
| Single | 25 (19.5) | 15 (20.0) | 10 (18.9) |  |
| Married | 68 (53.1) | 36 (48.0) | 32 (60.4) |  |
| Registered partner | 1 (0.8) | 0 (0.0) | 1 (1.9) |  |
| Divorced | 24 (18.8) | 15 (20.0) | 9 (17.0) |  |
| Widowed | 9 (7.0) | 8 (10.7) | 1 (1.9) |  |
| Missing data | 1 (0.8) | 1 (1.3) | 0 (0.0) |  |
| **Household income (%)** |  |  |  | 0.106 |
| Q1 | 25 (19.5) | 19 (25.3) | 6 (11.3) |  |
| Q2 | 37 (28.9) | 24 (32.0) | 13 (24.5) |  |
| Q3 | 34 (26.6) | 16 (21.3) | 18 (34.0) |  |
| Q4 | 31 (24.2) | 15 (20.0) | 16 (30.2) |  |
| Missing data | 1 (0.8) | 1 (1.3) | 0 (0.0) |  |
| **Educational level (%)** |  |  |  | 0.344 |
| <High school | 1 (0.8) | 1 (1.3) | 0 (0.0) |  |
| High school | 27 (21.1) | 19 (25.3) | 8 (15.1) |  |
| Higher education | 44 (34.4) | 26 (34.7) | 18 (34.0) |  |
| Missing data | 56 (43.8) | 29 (38.7) | 27 (50.9) |  |
| **Employment (%)** |  |  |  | 0.279 |
| Employed | 75 (58.6) | 40 (53.3) | 35 (66.0) |  |
| Unemployed | 52 (40.6) | 34 (45.3) | 18 (34.0) |  |
| Missing data | 1 (0.8) | 1 (1.3) | 0 (0.0) |  |

Abbreviations: CNS, central nervous system; EU28, European Union with all 28 member states; Q, Quartile

P-values were calculated using Chi-square test for categorical variables (with continuity correction) and ANOVA for continuous variables.

# Table S5

| **Patient and tumor characteristics of triple-negative breast cancer patients (n = 5846), stratified by time to relapse or death for the sensitivity analysis (healthcare regions with no available relapse data were excluded)** | | | | | |
| --- | --- | --- | --- | --- | --- |
|  |  |  |  |  |  |
| **Characteristic** | **Overall (n = 5846)** | **Rapid relapse or death (n = 905)** | **Late relapse or death (n = 1049)** | **Event-free at last follow-up (n = 3892)** | **P** |
| **Swedish region (%)** |  |  |  |  | 0.205 |
| Stockholm/Gotland | 1870 (32.0) | 303 (33.5) | 309 (29.5) | 1258 (32.3) |  |
| South | 1534 (26.2) | 224 (24.8) | 267 (25.5) | 1043 (26.8) |  |
| Southeast | 825 (14.1) | 127 (14.0) | 153 (14.6) | 545 (14.0) |  |
| West | 1617 (27.7) | 251 (27.7) | 320 (30.5) | 1046 (26.9) |  |
| **Age, years, median [IQR]** | 61.0 [49.0, 72.0] | 70.0 [53.0, 82.0] | 69.0 [57.0, 79.0] | 58.0 [47.0, 67.0] | **<0.001** |
| **Age group, years (%)** |  |  |  |  | **<0.001** |
| <40 | 597 (10.2) | 70 (7.7) | 63 (6.0) | 464 (11.9) |  |
| 40-49 | 926 (15.8) | 114 (12.6) | 101 (9.6) | 711 (18.3) |  |
| 50-64 | 1869 (32.0) | 176 (19.4) | 264 (25.2) | 1429 (36.7) |  |
| 65-74 | 1310 (22.4) | 159 (17.6) | 239 (22.8) | 912 (23.4) |  |
| ≥75 | 1144 (19.6) | 386 (42.7) | 382 (36.4) | 376 (9.7) |  |
| **Clinical stage (%)** |  |  |  |  | **<0.001** |
| 0 | 29 (0.5) | 0 (0.0) | 2 (0.2) | 27 (0.7) |  |
| IA | 2156 (36.9) | 124 (13.7) | 324 (30.9) | 1708 (43.9) |  |
| IIA | 2088 (35.7) | 320 (35.4) | 406 (38.7) | 1362 (35.0) |  |
| IIB | 869 (14.9) | 266 (29.4) | 172 (16.4) | 431 (11.1) |  |
| IIIA | 202 (3.5) | 90 (9.9) | 37 (3.5) | 75 (1.9) |  |
| IIIB | 100 (1.7) | 60 (6.6) | 24 (2.3) | 16 (0.4) |  |
| IIIC | 35 (0.6) | 15 (1.7) | 8 (0.8) | 12 (0.3) |  |
| Unspecified | 367 (6.3) | 30 (3.3) | 76 (7.2) | 261 (6.7) |  |
| **Tumor size, mm, median [IQR]*** | 30.0 [22.0, 40.0] | 40.0 [26.3, 50.0] | 35.5 [31.3, 48.0] | 27.5 [21.0, 35.8] | **<0.001** |
| **Ki67% (mean (SD))*** | 64.3 (23.6) | 62.7 (24.5) | 60.5 (25.0) | 65.4 (23.0) | **0.043** |
| **Pathological stage (%)†** |  |  |  |  | **<0.001** |
| 0 | 21 (0.4) | 1 (0.1) | 0 (0.0) | 20 (0.5) |  |
| IA | 1872 (32.0) | 100 (11.0) | 235 (22.4) | 1537 (39.5) |  |
| IIA | 1653 (28.3) | 174 (19.2) | 314 (29.9) | 1165 (29.9) |  |
| IIB | 654 (11.2) | 140 (15.5) | 146 (13.9) | 368 (9.5) |  |
| IIIA | 410 (7.0) | 163 (18.0) | 111 (10.6) | 136 (3.5) |  |
| IIIC | 176 (3.0) | 101 (11.2) | 38 (3.6) | 37 (1.0) |  |
| Missing data | 1060 (18.1) | 226 (25.0) | 205 (19.5) | 629 (16.2) |  |
| **Cancer type (%)†** |  |  |  |  | **<0.001** |
| TNBC-NST | 4041 (69.1) | 559 (61.8) | 760 (72.4) | 2722 (69.9) |  |
| Other invasive cancer | 675 (11.5) | 82 (9.1) | 120 (11.4) | 473 (12.2) |  |
| TNBC-ILC | 121 (2.1) | 25 (2.8) | 33 (3.1) | 63 (1.6) |  |
| Both TNBC-NST and TNBC-ILC | 23 (0.4) | 5 (0.6) | 4 (0.4) | 14 (0.4) |  |
| TNBC-NST and other type than TNBC-ILC | 74 (1.3) | 10 (1.1) | 13 (1.2) | 51 (1.3) |  |
| Missing data | 912 (15.6) | 224 (24.8) | 119 (11.3) | 569 (14.6) |  |
| **Tumor size, mm, median [IQR]†** | 20.0 [13.0, 29.0] | 30.0 [19.0, 45.0] | 22.0 [15.0, 32.0] | 17.0 [11.0, 25.0] | **<0.001** |
| **NHG (%)†** |  |  |  |  | **<0.001** |
| Grade 1 | 123 (2.1) | 6 (0.7) | 24 (2.3) | 93 (2.4) |  |
| Grade 2 | 1000 (17.1) | 110 (12.2) | 206 (19.6) | 684 (17.6) |  |
| Grade 3 | 3775 (64.6) | 560 (61.9) | 689 (65.7) | 2526 (64.9) |  |
| **Ki67% (mean (SD))†** | 56.9 (27.5) | 61.0 (25.2) | 56.0 (26.4) | 56.2 (28.0) | **0.002** |
| **Time to distant relapse or death (mean (SD))** | 77.3 (51.5) | 13.4 (6.4) | 63.7 (36.1) | 95.8 (47.8) | **<0.001** |
| **Number of distant metastatic sites (%)** |  |  |  |  | **<0.001** |
| 0 | 5108 (87.4) | 445 (49.2) | 771 (73.5) | 3892 (100.0) |  |
| 1 | 456 (7.8) | 292 (32.3) | 164 (15.6) | 0 (0.0) |  |
| 2 | 192 (3.3) | 120 (13.3) | 72 (6.9) | 0 (0.0) |  |
| ≥3 | 90 (1.5) | 48 (5.3) | 42 (4.0) | 0 (0.0) |  |
| **Comorbidities (%)** |  |  |  |  |  |
| Myocardial infarction | 127 (2.2) | 43 (4.8) | 44 (4.2) | 40 (1.0) | **<0.001** |
| Congestive heart failure | 116 (2.0) | 52 (5.7) | 34 (3.2) | 30 (0.8) | **<0.001** |
| Peripheral vascular disease | 106 (1.8) | 37 (4.1) | 25 (2.4) | 44 (1.1) | **<0.001** |
| Cerebrovascular disease | 299 (5.1) | 75 (8.3) | 79 (7.5) | 145 (3.7) | **<0.001** |
| Chronic obstructive pulmonary disease | 96 (1.6) | 27 (3.0) | 31 (3.0) | 38 (1.0) | **<0.001** |
| Chronic other pulmonary disease | 188 (3.2) | 29 (3.2) | 41 (3.9) | 118 (3.0) | 0.36 |
| Rheumatic disease | 253 (4.3) | 55 (6.1) | 55 (5.2) | 143 (3.7) | **0.002** |
| Dementia | 43 (0.7) | 26 (2.9) | 13 (1.2) | 4 (0.1) | **<0.001** |
| Hemiplegia | 16 (0.3) | 3 (0.3) | 3 (0.3) | 10 (0.3) | 0.925 |
| Diabetes without chronic complication | 6 (0.1) | 1 (0.1) | 0 (0.0) | 5 (0.1) | 0.513 |
| Diabetes with chronic complication | 91 (1.6) | 19 (2.1) | 24 (2.3) | 48 (1.2) | **0.018** |
| Renal disease | 49 (0.8) | 18 (2.0) | 9 (0.9) | 22 (0.6) | **<0.001** |
| Mild liver disease | 51 (0.9) | 11 (1.2) | 14 (1.3) | 26 (0.7) | 0.058 |
| Liver special | 5 (0.1) | 1 (0.1) | 1 (0.1) | 3 (0.1) | 0.946 |
| Severe liver disease | 5 (0.1) | 0 (0.0) | 2 (0.2) | 3 (0.1) | 0.339 |
| Peptic ulcer disease | 91 (1.6) | 22 (2.4) | 31 (3.0) | 38 (1.0) | **<0.001** |
| Malignancy | 747 (12.8) | 142 (15.7) | 191 (18.2) | 414 (10.6) | **<0.001** |
| Metastatic solid tumor | 50 (0.9) | 12 (1.3) | 14 (1.3) | 24 (0.6) | **0.02** |
| AIDS | 3 (0.1) | 1 (0.1) | 1 (0.1) | 1 (0.0) | 0.469 |
| **CCIw group (%)** |  |  |  |  | **<0.001** |
| CCIw=0 | 4101 (70.2) | 524 (57.9) | 618 (58.9) | 2959 (76.0) |  |
| CCIw=1-3 | 1591 (27.2) | 331 (36.6) | 389 (37.1) | 871 (22.4) |  |
| CCIw=4-10 | 154 (2.6) | 50 (5.5) | 42 (4.0) | 62 (1.6) |  |

Abbreviations: CCIw, weighted Charlson comorbidity index; TNBC, Triple-negative breast cancer; TNBC–ILC, Triple-negative breast cancer–invasive lobular cancer; TNBC–NST, Triple-negative breast cancer of no special type. Statistically significant differences (P < 0.05) are highlighted in bold. P-values were calculated using Chi-square test for categorical variables (with continuity correction) and ANOVA for continuous variables.

# Table S6

| **Treatment characteristics of triple-negative breast cancer patients (n = 5846), stratified by time to relapse or death for the sensitivity analysis (healthcare regions with no available relapse data were excluded)** | | | | | |
| --- | --- | --- | --- | --- | --- |
|  |  |  |  |  |  |
| **Characteristic** | **Overall (n =5846)** | **Rapid relapse or death (n = 905)** | **Late relapse or death (n = 1049)** | **Event-free at last follow-up (n = 3892)** | **P** |
| **NACT type (%)** |  |  |  |  | **<0.001** |
| No NACT | 17 (0.3) | 4 (0.4) | 8 (0.8) | 5 (0.1) |  |
| Anthracycline-based | 222 (3.8) | 61 (6.7) | 41 (3.9) | 120 (3.1) |  |
| Anthracycline+taxane | 912 (15.6) | 145 (16.0) | 82 (7.8) | 685 (17.6) |  |
| Taxane-based | 60 (1.0) | 22 (2.4) | 6 (0.6) | 32 (0.8) |  |
| Other | 23 (0.4) | 6 (0.7) | 4 (0.4) | 13 (0.3) |  |
| Missing data | 4612 (78.9) | 667 (73.7) | 908 (86.6) | 3037 (78.0) |  |
| **Axillary surgery type (%)** |  |  |  |  | **<0.001** |
| SLNB | 3355 (57.4) | 222 (24.5) | 493 (47.0) | 2640 (67.8) |  |
| AD | 1435 (24.5) | 416 (46.0) | 308 (29.4) | 711 (18.3) |  |
| Sampling (including SLNB) | 89 (1.5) | 11 (1.2) | 21 (2.0) | 57 (1.5) |  |
| SLNB + AD | 680 (11.6) | 108 (11.9) | 149 (14.2) | 423 (10.9) |  |
| **Surgery type (%)** |  |  |  |  | **<0.001** |
| Only axillary surgery | 21 (0.4) | 1 (0.1) | 2 (0.2) | 18 (0.5) |  |
| Breast-conserving surgery | 3027 (51.8) | 216 (23.9) | 414 (39.5) | 2397 (61.6) |  |
| Mastectomy | 2624 (44.9) | 592 (65.4) | 609 (58.1) | 1423 (36.6) |  |
| Subcutaneous mastectomy | 52 (0.9) | 6 (0.7) | 3 (0.3) | 43 (1.1) |  |
| Missing data | 122 (2.1) | 90 (9.9) | 21 (2.0) | 11 (0.3) |  |
| **Locoregional treatment (%)** |  |  |  |  | **<0.001** |
| BCS+ART | 2469 (42.2) | 127 (14.0) | 288 (27.5) | 2054 (52.8) |  |
| Mx-ART | 1135 (19.4) | 217 (24.0) | 301 (28.7) | 617 (15.9) |  |
| Mx+ART | 1034 (17.7) | 241 (26.6) | 201 (19.2) | 592 (15.2) |  |
| Other | 1208 (20.7) | 320 (35.4) | 259 (24.7) | 629 (16.2) |  |
| **ACT type (%)** |  |  |  |  | **<0.001** |
| No ACT | 1843 (31.5) | 350 (38.7) | 411 (39.2) | 1082 (27.8) |  |
| Anthracycline-based | 1288 (22.0) | 91 (10.1) | 229 (21.8) | 968 (24.9) |  |
| Anthracycline+taxane | 1174 (20.1) | 73 (8.1) | 99 (9.4) | 1002 (25.7) |  |
| Taxane-based | 176 (3.0) | 40 (4.4) | 17 (1.6) | 119 (3.1) |  |
| Other | 458 (7.8) | 97 (10.7) | 101 (9.6) | 260 (6.7) |  |
| Missing data | 907 (15.5) | 254 (28.1) | 192 (18.3) | 461 (11.8) |  |
| **ART (%)** | 3560 (60.9) | 380 (42.0) | 499 (47.6) | 2681 (68.9) | **<0.001** |
| **Adjuvant bisphosphonate therapy (%)** | 680 (11.6) | 62 (6.9) | 75 (7.1) | 543 (14.0) | **<0.001** |

Abbreviations: ACT, adjuvant chemotherapy; AD, axillary dissection; ART, adjuvant radiotherapy; BCS, breast-conserving surgery; Mx, mastectomy; NACT, neoadjuvant chemotherapy; SLNB, sentinel lymph-node biopsy. Statistically significant differences (P < 0.05) are highlighted in bold. P-values were calculated using Chi-square test for categorical variables (with continuity correction) and ANOVA for continuous variables.

# Table S7

| **Sociodemographic features of triple-negative breast cancer patients (n = 5846), stratified by time to relapse or death for the sensitivity analysis (healthcare regions with no available relapse data were excluded)** | | | | | |
| --- | --- | --- | --- | --- | --- |
|  |  |  |  |  |  |
| **Characteristic** | **Overall (n = 5846)** | **Rapid relapse or death (n = 905)** | **Late relapse or death (n = 1049)** | **Event-free at last follow-up (n = 3892)** | **P** |
| **Patient's birth country (%)** |  |  |  |  | **<0.001** |
| Sweden | 4785 (81.9) | 717 (79.2) | 881 (84.0) | 3187 (81.9) |  |
| Africa | 53 (0.9) | 8 (0.9) | 11 (1.0) | 34 (0.9) |  |
| Asia | 233 (4.0) | 30 (3.3) | 26 (2.5) | 177 (4.5) |  |
| EU28, non-Nordic countries | 259 (4.4) | 47 (5.2) | 38 (3.6) | 174 (4.5) |  |
| Europe, non-EU28 and Nordic countries | 154 (2.6) | 25 (2.8) | 19 (1.8) | 110 (2.8) |  |
| North America | 20 (0.3) | 6 (0.7) | 2 (0.2) | 12 (0.3) |  |
| Nordic countries, not Sweden | 274 (4.7) | 56 (6.2) | 58 (5.5) | 160 (4.1) |  |
| Oceania | 1 (0.0) | 0 (0.0) | 0 (0.0) | 1 (0.0) |  |
| Former Soviet Union | 4 (0.1) | 0 (0.0) | 0 (0.0) | 4 (0.1) |  |
| South America | 39 (0.7) | 6 (0.7) | 4 (0.4) | 29 (0.7) |  |
| Missing data | 24 (0.4) | 10 (1.1) | 10 (1.0) | 4 (0.1) |  |
| **Father's birth country (%)** |  |  |  |  | **<0.001** |
| Sweden | 3793 (64.9) | 461 (50.9) | 541 (51.6) | 2791 (71.7) |  |
| Africa | 4 (0.1) | 0 (0.0) | 0 (0.0) | 4 (0.1) |  |
| Asia | 21 (0.4) | 2 (0.2) | 3 (0.3) | 16 (0.4) |  |
| EU28, non-Nordic countries | 101 (1.7) | 11 (1.2) | 12 (1.1) | 78 (2.0) |  |
| Europe, non-EU28 and Nordic countries | 45 (0.8) | 7 (0.8) | 3 (0.3) | 35 (0.9) |  |
| North America | 5 (0.1) | 0 (0.0) | 0 (0.0) | 5 (0.1) |  |
| Nordic countries, not Sweden | 141 (2.4) | 14 (1.5) | 22 (2.1) | 105 (2.7) |  |
| Former Soviet Union | 4 (0.1) | 1 (0.1) | 0 (0.0) | 3 (0.1) |  |
| South America | 9 (0.2) | 0 (0.0) | 2 (0.2) | 7 (0.2) |  |
| Missing data | 1723 (29.5) | 409 (45.2) | 466 (44.4) | 848 (21.8) |  |
| **Mother's birth country (%)** |  |  |  |  | **<0.001** |
| Sweden | 3879 (66.4) | 464 (51.3) | 569 (54.2) | 2846 (73.1) |  |
| Africa | 3 (0.1) | 0 (0.0) | 0 (0.0) | 3 (0.1) |  |
| Asia | 27 (0.5) | 1 (0.1) | 5 (0.5) | 21 (0.5) |  |
| EU28, non-Nordic countries | 103 (1.8) | 15 (1.7) | 10 (1.0) | 78 (2.0) |  |
| Europe, non-EU28 and Nordic countries | 48 (0.8) | 7 (0.8) | 3 (0.3) | 38 (1.0) |  |
| North America | 9 (0.2) | 1 (0.1) | 2 (0.2) | 6 (0.2) |  |
| Nordic countries, not Sweden | 207 (3.5) | 29 (3.2) | 33 (3.1) | 145 (3.7) |  |
| Former Soviet Union | 4 (0.1) | 0 (0.0) | 0 (0.0) | 4 (0.1) |  |
| South America | 14 (0.2) | 2 (0.2) | 1 (0.1) | 11 (0.3) |  |
| Missing data | 1552 (26.5) | 386 (42.7) | 426 (40.6) | 740 (19.0) |  |
| **Marital status (%)** |  |  |  |  | **<0.001** |
| Single | 1042 (17.8) | 128 (14.1) | 144 (13.7) | 770 (19.8) |  |
| Married | 2841 (48.6) | 329 (36.4) | 460 (43.9) | 2052 (52.7) |  |
| Registered partner | 2 (0.0) | 0 (0.0) | 1 (0.1) | 1 (0.0) |  |
| Divorced | 1080 (18.5) | 142 (15.7) | 182 (17.3) | 756 (19.4) |  |
| Widowed | 768 (13.1) | 211 (23.3) | 252 (24.0) | 305 (7.8) |  |
| Missing data | 113 (1.9) | 95 (10.5) | 10 (1.0) | 8 (0.2) |  |
| **Household income (%)** |  |  |  |  | **<0.001** |
| Q1 | 1436 (24.6) | 300 (33.1) | 381 (36.3) | 755 (19.4) |  |
| Q2 | 1352 (23.1) | 220 (24.3) | 256 (24.4) | 876 (22.5) |  |
| Q3 | 1412 (24.2) | 158 (17.5) | 198 (18.9) | 1056 (27.1) |  |
| Q4 | 1533 (26.2) | 132 (14.6) | 204 (19.4) | 1197 (30.8) |  |
| Missing data | 113 (1.9) | 95 (10.5) | 10 (1.0) | 8 (0.2) |  |
| **Educational level (%)** |  |  |  |  | **<0.001** |
| <High school | 1176 (20.1) | 242 (26.7) | 319 (30.4) | 615 (15.8) |  |
| High school | 2381 (40.7) | 308 (34.0) | 412 (39.3) | 1661 (42.7) |  |
| Higher education | 2121 (36.3) | 246 (27.2) | 299 (28.5) | 1576 (40.5) |  |
| Missing data | 168 (2.9) | 109 (12.0) | 19 (1.8) | 40 (1.0) |  |
| **Employment (%)** |  |  |  |  | **<0.001** |
| Employed | 2794 (47.8) | 266 (29.4) | 308 (29.4) | 2220 (57.0) |  |
| Unemployed | 2931 (50.1) | 543 (60.0) | 731 (69.7) | 1657 (42.6) |  |
| Missing data | 121 (2.1) | 96 (10.6) | 10 (1.0) | 15 (0.4) |  |

Abbreviations: EU28, European Union with all 28 member states; Q, Quartile

P-values were calculated using Chi-square test for categorical variables (with continuity correction) and ANOVA for continuous variables.
